# Electrospun Nanofiber Therapeutics for Localized Post-Surgical Breast Cancer Treatment

**DOI:** 10.3390/jfb17100489

**Published:** 2026-09-28

**Authors:** Elmira Sadat Tabatabaei, Ozge Sagir, Morgan Bretches, David Long, Shang-You Yang, Eylem Asmatulu

**Affiliations:** 1 Department of Biomedical Engineering, Wichita State University, Wichita, KS 67260, USA; extabatabaei@shockers.wichita.edu (E.S.T.); david.long@wichita.edu (D.L.); 2 Department of Mechanical Engineering, Wichita State University, Wichita, KS 67260, USA; oxsagir@shockers.wichita.edu; 3 Department of Orthopedic Surgery, University of Kansas School of Medicine, Wichita, KS 67214, USA; mbretches@kumc.edu; 4 Department of Biological Sciences, Wichita State University, Wichita, KS 67260, USA

**Keywords:** cancer therapy, breast cancer, electrospun nanofibers, localized drug delivery, controlled release

## Abstract

Adjuvant therapy following breast cancer surgery remains challenged by systemic toxicity, poor tumor accuracy, and tumor recurrence. Electrospun nanofibers have emerged to address these concerns. Their high surface area, tunable porosity, and controlled drug release properties enable sustained, site-specific drug release directly to the tumor site while sparing healthy tissues and reducing systemic exposure. The scientific question of the current review is centered on whether the utilization of electrospun nanofibers prevents tumor recurrence after breast cancer surgery. Electrospun nanofiber design determinants, including composition, architecture, creation techniques, and drug-loading strategies, are outlined in the following analysis. Subsequently, it also examines different therapeutic approaches, such as protein- and peptide-loaded fibers, stimuli-responsive nanofibers, natural anticancer compounds, immunomodulators, and combination therapies. Notably, the application of electrospun nanofibers in breast cancer treatment, overcoming tumor microenvironment barriers, and preventing post-surgical recurrence is reported. Finally, limitations associated with clinical translation, such as large-scale production, regulatory approval, sterilization, and long-term efficacy, are also addressed. Collectively, the evidence indicates that electrospun nanofibers show great potential for localized cancer therapy by enabling controlled drug release and reducing systemic exposure, though clinical validation remains the critical unmet step.

## 1. Introduction

The American Cancer Society predicts more than 2.1 million new cancer diagnoses in the United States in 2026, or about 5800 cases per day [1]. Cancer develops from a single cell that begins to replicate uncontrollably and invade surrounding tissues. Breast cancer is one of the most common types of cancer, and even when it spreads to other parts of the body, it remains classified as breast cancer based on its tissue of origin [2].

Despite significant clinical advances in cancer treatment, traditional systemic therapies still have several limitations. Standard treatment modalities, including chemotherapy, radiation therapy, hormone therapy, and targeted therapy, are frequently associated with significant adverse effects. When delivered systemically, anticancer agents distribute indiscriminately rather than solely at the tumor site, resulting in off-target damage to healthy cells. More than seventy conventional chemotherapy drugs have been approved by the FDA, but many are associated with serious side effects, including damage to cardiac tissue, reduced blood cell counts, hair loss, fertility problems, and digestive complications [3,4,5,6]. Tumors commonly evolve resistance to treatment through several mechanisms, including drug efflux by P-glycoprotein and increased detoxification [4,5,6]. Moreover, tumors can survive radiation therapy when cancer cells reside in low-oxygen (hypoxic) regions or when they constitute a substantial population of tumor stem-like cells, which can reduce the efficiency of the treatment and allow continued tumor growth. Hypoxia-inducible factor-1α (HIF-1α) has been shown to play an important role in treatment resistance, particularly in triple-negative breast cancer [7]. Additionally, most of the anticancer drugs remain in the body for a limited time, leading to reduce the amount of drug which reaches the tumor site and may contribute to have the lower effect of the treatment, increasing the risk of recurrence. Therefore, localized drug delivery systems are being studied to deliver therapeutic agents directly to the tumor site, extend drug release, and reduce damage to healthy tissues.

Epidemic data on breast cancer highlights a continued need for more effective treatment strategies. In 2024, breast cancer was the most diagnosed cancer type among women, with an estimated 310,720 cases in women and 2790 cases in men. Breast cancer risk is affected by genetic, hormonal, and environmental factors, including age, early menstruation, late menopause, no pregnancy history, family history, dense breast tissue, and BRCA1/BRCA2 mutations [8]. The disease is classified according to tumor size, lymph node involvement, metastasis, and molecular markers such as ER, PR, and HER2/ERBB2. Tumors that do not express these three receptors are called triple-negative breast cancers (TNBCs), which are associated with poor prognosis and limited targeted treatment options. Treatment methods such as surgery, radiotherapy, chemotherapy, and targeted therapy are commonly used and can be effective in early-stage disease [9]. However, these methods still have important limitations, and local recurrence can occur after breast-conserving surgery. In addition, metastatic breast cancer (MBC) affects about 168,000 women in the United States, including both newly diagnosed and recurrent cases [10]. Despite technical improvements designed to protect healthy tissues, radiotherapy and chemotherapy are still associated with toxicity, treatment resistance, and possible treatment failure [11,12,13,14]. Therefore, localized and precision drug delivery systems are being developed to concentrate therapeutic agents at the tumor site, provide controlled and sustained release, and reduce systemic exposure, improving treatment efficiency, survival, and quality of life for breast cancer patients.

Advances in nanotechnology have positively impacted cancer treatment by enhancing the solubility and stabilization of medicines in addition to protecting drugs from being broken down prematurely. The use of nanomaterials will also improve drug localization in the tumor while sparing the healthy cells from destruction [15]. Various approaches have been investigated for drug delivery according to nanotechnology, such as dendrimers, liposomes, polymeric micelles, nanocapsules, etc. [3,16]. Of these, electrospun nanofibers are unique in enabling precise drug release confined to the treatment site [17]. Electrospun nanofibers have a high surface-area-to-volume ratio and adjustable porous structure, supporting efficient drug loading and controlled drug release. These fibers can also mimic the extracellular matrix (ECM), which supports cell attachment and tissue integration [18]. However, these properties should be carefully controlled in a post-surgical tumor bed because they may also influence residual cancer cell behavior. Electrospun nanofibers can be produced with synthetic, natural, and hybrid polymers, and may also incorporate fragile molecules such as peptides, proteins, and immunomodulators [19].

Advanced methods such as coaxial, triaxial, and emulsion electrospinning can produce core–shell, multilayered, or core–shell-like fiber architectures that protect sensitive bioactive molecules and provide controlled release [20,21,22,23]. In breast cancer treatment, nanofiber mats can be placed into the surgical area as implants to release therapeutic agents directly to the affected area. Therefore, these systems may help reduce recurrence risk and systemic exposure [20,22]. Due to their similarity to natural ECM, electrospun nanofibers are also promising materials for localized drug delivery.

Several reviews have discussed electrospun nanofibers for drug delivery and cancer treatment [16,24,25,26]. These reviews cover electrospinning methods, drug loading, release behavior, and different cancer applications. However, most of them discuss cancer therapy in a broader way. A focused review of electrospun systems for localized treatment after breast cancer surgery is still limited, which is the scope of the present review. It discusses the design of electrospun systems for the post-surgical breast tumor bed, including drug release, tumor microenvironment, surgical handling, fixation, sterilization, scale-up, and clinical translation.

This review focuses on electrospun nanofibers as localized and precision drug delivery systems for breast cancer treatment. It discusses the basic principles and configurations of electrospinning used in biomedical applications together with material selection, fiber architecture, and the main factors affecting drug encapsulation and release. Different encapsulation strategies for chemotherapeutic drugs, peptides, proteins, natural agents, immunomodulators, and combination therapies are compared. In addition, characterization results reported in previous studies, including morphology, chemical composition, mechanical properties, and biological evaluation, are reviewed.

Specific attention is given to breast cancer therapy, especially post-surgical recurrence prevention. Tumor microenvironment barriers and engineering strategies used in nanofiber systems to overcome these barriers are also examined. Stimuli-responsive electrospun systems, including pH-, ROS-, thermal-, and light-responsive nanofibers, are discussed together with smart and multifunctional fiber designs.

Furthermore, translational, regulatory, and manufacturing challenges are evaluated. Clinical translation is discussed with special attention to future clinical trials, personalized therapy, and model-driven treatment approaches. Overall, this review links electrospinning design parameters and drug delivery performance with the specific requirements of the post-surgical breast tumor bed, including local retention, release duration, biodegradation, sterilization compatibility, and surgical handling.

This review was prepared in the form of a structured narrative. Previous studies related to electrospun nanofibers, drug delivery, cancer therapy, and breast cancer were searched in scientific databases. The search used keywords such as “electrospinning,” “electrospun nanofibers,” “drug delivery,” “breast cancer,” “localized cancer therapy,” “post-surgical treatment,” and “cancer recurrence.” Studies were selected based on their relation to the electrospinning method, materials, drug loading, drug release, breast cancer application, tumor microenvironment, and clinical translation. Primary research articles and recent review articles were primarily included. Some older studies were also included to provide basic information about electrospinning methods and/or drug delivery. The literature included has studies published from the year 1994 to 2026.

## 2. Fundamentals of Electrospinning for Drug Delivery

Electrospinning is a widely used technique for fabricating ultrafine fibers with diameters ranging from tens of nanometers to several micrometers. This approach is particularly valuable in drug delivery applications, as it produces highly porous fibers with a large surface area. Therapeutic agents can be incorporated into electrospun fibers, enabling controlled and sustained drug release. Moreover, electrospun fiber matrices can closely replicate the structure of the extracellular matrix, making them highly suitable for a variety of biomedical applications.

### 2.1. Basic Principles

Electrospinning depends on the interaction between electrostatic forces, fluid behavior, and solvent evaporation. Ramakrishna et al. reported that a polymer solution is pushed through a spinneret and a high voltage is applied to charge the solution. When the electrostatic repulsion becomes stronger than the surface tension, a Taylor cone is formed at the needle tip. Then, a charged polymer jet is ejected from the tip and moves toward a grounded collector, as shown in Figure 1. During this movement, the jet is stretched and thinned, while the solvent evaporates. As a result, solid fibers are formed and collected on the collector surface [27,28].

The process is based on several important mechanisms. Electrostatic stretching helps the charged polymer chains to elongate and reach nanoscale fiber diameters. Bending instability causes the jet to move in a whipping path, and this makes the fibers thinner before they are collected. Solid fibers are formed when the solvent evaporates. At the same time, polymer entanglements prevent the jet from breaking and help continuous fibers to be obtained. The grounded collector, which is usually a flat plate or rotating drum, affects the way fibers are collected. In general, polymers with suitable molecular weight and solubility can be electrospun. Polymer blends and drug-loaded polymer formulations can also be produced by electrospinning.

### 2.2. Key Process Parameters

Electrospinning can be adjusted precisely, and fiber shape and structure are affected by several connected parameters. The main electrospinning parameters can be grouped into environmental, formulation, and device-related factors, as shown in Figure 2.

Solution properties are important in determining fiber formation and morphology. Viscosity and polymer concentration should be high enough to maintain chain entanglement. Low viscosity can cause bead defects, as shown by the beaded morphology in Figure 3A. Conductivity affects the stretching behavior of the jet, and higher conductivity increases the charge-carrying capacity. Solvent volatility also affects the solvent evaporation rate. High volatility can promote porous fiber formation through phase separation [23,29].

Processing conditions also play an important role in electrospinning. Applied voltage is necessary for initiating the Taylor cone and sustaining the jet. When the voltage is too low, spinning may not occur. On the other hand, very high voltage can destabilize the jet. Flow rate controls the jet thickness. Excess flow rates can lead to bead formation, while lower flow rates can produce thinner fibers. In addition, the distance between the needle and the collector should be sufficient to allow solvent evaporation [30]. Different fiber morphologies obtained under different conditions are presented in Figure 3.

Collector design affects fiber deposition and final structure. Static collectors generally produce nonwoven meshes, which are the most common structures. Rotating mandrels and auxiliary electrodes generate aligned, patterned, or tubular fiber assemblies. As shown in Figure 4, electrospun fibers can be collected as random two-dimensional fibrous meshes, aligned two-dimensional structures, or three-dimensional structures depending on the collector design. Fiber alignment can also be controlled by changing the electric field geometry and mechanical rotation, as shown in Figure 5. Fiber orientation can be controlled by manipulating electric-field geometry and collector motion. Such control can improve the mechanical properties and structural organization of electrospun fibers [31,32,33,34].

### 2.3. Considerations for Drug Encapsulation and Release

Electrospinning is an effective method for drug delivery because therapeutic agents can be directly incorporated into polymer matrices, and tunable release profiles can be obtained. Previous studies have shown that encapsulation efficiency and release kinetics are affected by several factors [19,35,36].

Encapsulation Strategies: Different encapsulation strategies are available in electrospinning. In blend electrospinning, the drug is mixed uniformly with the polymer solution. In core–shell electrospinning, a dual-needle system produces fibers with the drug in the core and a protective polymer shell. FITC-BSA encapsulated in PCL core–shell fibers have shown sustained release over several days, as shown in Figure 6.

Drug Stability: Electrospinning is generally performed at room temperature, and harsh processing conditions are avoided. For this reason, heat-sensitive compounds such as proteins, peptides, and growth factors can be encapsulated without significant degradation.

Release Mechanisms: Drug release profiles depend on the interaction between fiber structure and polymer properties. Diffusion-controlled release can occur when the drug is located near the fiber surface or when a water-soluble polymer matrix is used. Nevertheless, high drug localization adjacent to the fiber surface could lead to an early burst release, which is usually an issue in matrix-controlled systems and is dependent on the drug distribution, polymer properties, and diffusion routes [37]. Degradation-controlled release can be observed in biodegradable polymers such as PCL, PLLA, and PLGA. Cell-mediated release acceleration can also occur in these systems. Human dermal fibroblasts have been shown to increase FITC-BSA release due to enzymatic degradation of the polymer [28].

Fiber Morphology and Porosity: Porous fibers can be formed through solvent-induced phase separation. These fibers can increase the drug release rate because more water can penetrate the fiber structure. In addition, a higher surface area is exposed to the release medium.

Surface Functionalization: Nanofibers can be functionalized with biological ligands. This functionalization can provide targeted delivery through receptor-binding ligands, controlled cell interaction, and biosensing. Ramakrishna et al. demonstrated that nanofiber scaffolds can be conjugated with ligands for cell-specific targeting and controlled insulin delivery [28,38,39].

## 3. Electrospinning Technologies for Cancer Therapy Applications

Electrospinning provides different fabrication strategies that can control fiber shape, drug encapsulation, and release behavior. These properties make electrospun nanofibers important for cancer therapy applications. In this field, localized delivery, biomolecule stability, and adjustable release behavior are needed. In the following subsections, different electrospinning configurations and their use in cancer drug delivery are discussed, especially for the encapsulation of chemotherapeutics, peptides, proteins, and other therapeutic macromolecules [19]. Figure 7 shows that biomedical nanofibers can be fabricated using several electrospinning configurations, including single-nozzle, coaxial, triaxial, and emulsion electrospinning.

### 3.1. Single-Nozzle Electrospinning

Single-nozzle electrospinning is one of the most commonly used techniques. It is easy to operate and works with many polymer systems. In this configuration, a polymer solution or melt is pushed through a charged metallic needle toward a grounded collector. When the electric field overcomes the surface tension, a Taylor cone is formed, and a continuous jet is produced. Then, this jet is solidified into fibers by solvent evaporation or cooling [40].

Fiber properties such as alignment, surface morphology, and porosity can be adjusted by changing collector design, environmental conditions, and solvent properties. For example, rotating drums or parallel electrodes help control fiber alignment, while humidity and temperature can affect fiber morphology. Therefore, single-nozzle electrospinning is suitable for producing drug-loaded sheets, wound dressings, and localized therapeutic mats for cancer applications [41].

Sensitive biomolecules such as peptides, proteins, and enzymes may also be denatured when they are exposed to organic solvents and high voltage. Side-by-side or Janus electrospinning can reduce some of these limitations. In this method, two parallel syringes are used to produce fibers with two different hemispheres, and this allows dual-drug loading. However, jet stability is still difficult to control, which limits the widespread use of this configuration [42].

### 3.2. Coaxial Electrospinning

Coaxial electrospinning is performed by using a concentric dual-needle spinneret. In this method, two different solutions flow at the same time, and core–shell nanofibers are formed. The shell solution stabilizes the jet and covers the core part. In this way, the therapeutic agent in the core can be separated from the external environment. For cancer therapy, coaxial electrospinning can preserve and protect sensitive biomolecules such as peptides, proteins, albumin, and enzymes from harsh solvents and high electric fields. Similar polymer-based encapsulation approaches have been developed for protein therapeutics, where protective carrier systems improve biomolecule stability and enable controlled release while preserving biological function [39]. High encapsulation efficiency can be achieved, and drug loss during fabrication can be minimized. The shell layer acts as a diffusion barrier, and this can provide sustained or zero-order release profiles. In addition, core solutions that are difficult to spin, especially hydrophilic formulations, can also be processed by this method. These properties make coaxial electrospinning useful for localized cancer therapy, especially when biomolecule stability and prolonged release are required. However, viscosity, flow rate, and solvent compatibility should be carefully controlled. If these parameters are not compatible, core leakage or jet instability can occur [35,36].

### 3.3. Triaxial Electrospinning

Triaxial electrospinning is developed from the coaxial electrospinning design by adding a third working fluid between the core and shell layers. In this method, three-layered fibers with more complex structures are produced. The additional layer acts as an extra barrier, and this allows better control of drug release behavior. Immediate, delayed, and sustained release phases can be obtained in a single fiber. In cancer therapy applications, triaxial electrospinning can support the sequential release of multiple therapeutic agents. For example, sensitizers can be released first, followed by chemotherapeutic drugs and targeting ligands. The increased number of encapsulation layers also provides better protection for fragile molecules. Therefore, combination therapy approaches can be developed within a single implantable platform. Despite their greater processing complexity, triaxial fibers are promising for cancer therapy, especially when time-controlled multi-agent delivery is needed [43,44].

### 3.4. Melt Electrospinning

Melt electrospinning uses a polymer melt instead of a polymer solution. In this way, the use of volatile or toxic solvents is eliminated. The molten polymer is charged and stretched into fibers under a temperature-controlled environment. Since no solvent is used, solvent residues are not left in the final fibers. This provides an important regulatory advantage for implantable cancer devices. Melt electrospinning can produce fibers with good mechanical strength and reproducibility. It also reduces the risk of solvent-related cytotoxicity. In addition, this method is not suitable for heat-sensitive therapeutic agents such as peptides, proteins, or small-molecule chemotherapeutics. Despite these limitations, melt electrospinning is still a useful method for producing implantable bioresorbable scaffolds and drug-eluting meshes used in postoperative cancer therapy [45,46,47].

### 3.5. Emulsion Electrospinning

Emulsion electrospinning uses water-in-oil or oil-in-water emulsions to produce fibers with core–shell-like morphology by using a single nozzle. In this method, hydrophilic drugs or proteins are kept inside dispersed aqueous droplets. During jet formation, these droplets are encapsulated in the polymer matrix. For cancer drug delivery, emulsion electrospinning is useful because hydrophilic biomolecules can be encapsulated effectively. Sustained release profiles similar to those of coaxial electrospinning can also be obtained. In addition, this method does not require special multi-needle equipment, which makes the process simpler. When the process is well optimized, emulsion electrospinning can be an effective method for the delivery of cancer-related peptides, proteins, and enzymes [48,49,50,51].

### 3.6. Needleless Electrospinning

Needleless electrospinning produces multiple jets directly from the surface of a polymer solution under a strong electric field. Different designs, such as rotating cylinders, rotating discs, wire coils, or bubble-assisted systems, can generate several jets at the same time. Therefore, the fiber production rate can significantly increase. This method is useful for large-area fabrication, industrial-scale production of drug-loaded mats, and reducing needle clogging problems. Needleless electrospinning is more suitable for transdermal patches, wound dressings, and protective cancer-related barriers. However, it is less suitable for precision-controlled drug delivery platforms that require detailed control of fiber architecture and release behavior [52,53,54].

## 4. Material Selection and Fiber Architectures

Electrospun drug-delivery systems are affected by several factors. Polymer selection is one of the most important factors because it influences mechanical behavior, degradation, and drug release performance. Fiber morphology and architecture also play an important role in the final performance of the system. These structures should have suitable mechanical properties and should degrade properly in the body.

Material selection generally includes natural, synthetic, or hybrid polymers. Fiber architecture also includes different structures such as smooth fibers, bead-on-string morphologies, and hierarchical structures. The relationship between structure and properties controls drug loading, mechanical strength, degradation behavior, and surgical handling. For this reason, these factors help determine whether the electrospun constructs are suitable as ECM-mimicking drug-delivery platforms in clinical applications.

A comparative overview of electrospinning techniques, including their key features, advantages, limitations, and biomedical applications, is presented in Table 1. Also, Representative configurations of these electrospinning techniques are illustrated in Figure 7.

### 4.1. Polymers: Natural vs. Synthetic

Electrospinning works with many different polymers. These polymers are generally classified as natural and synthetic polymers. Each group has its own advantages and limitations for biomedical and drug delivery applications, especially when nanofibers are designed to mimic the extracellular matrix (ECM).

Natural Polymers: Among the natural polymers that are used in electro-spinning are collagen, gelatin, chitosan, chitin, silk fibroin, and cellulose derivatives. These materials are actively studied for their use in biomedical applications due to their good biocompatibility and capability of supporting cell-material interactions. Collagen and gelatin in particular mimic biochemical features of the native extracellular matrix (ECM), and it should be noted that electrospun natural polymer fibers are able to replicate some features of native tissue fibrous architecture [65,66]. The hydrophilic character of these materials, and in some cases the presence of biologically active functional groups, helps them to improve cell adhesion, proliferation, and tissue integration. However, these advantages also come with some limitations [67,68]. Natural polymers generally have low mechanical strength, rapid or uncontrolled degradation, and limited electrospinnability. Gelatin and chitosan may dissolve or swell excessively in aqueous environments, and this can reduce scaffold integrity. To overcome these problems, natural polymer-based nanofibers are often blended with other polymers, such as gelatin with chitosan or chitosan with silk fibroin. Post-spinning crosslinking can also be applied to improve mechanical stability, control degradation, and maintain fibrous architecture under physiological conditions. For this reason, natural polymers provide good bioactivity, but reinforcement is usually needed for drug-loaded and implantable scaffolds [69].

Synthetic Polymers: Synthetic polymers such as polycaprolactone (PCL), poly(lactic acid) (PLA), poly(lactic-co-glycolic acid) (PLGA), polyurethane (PU), and nylon-6 are commonly used because they have good mechanical properties, predictable processing behavior, and tunable degradation [69]. These polymers can be electrospun easily, and uniform fibers can be obtained. The fiber diameter and morphology can also be controlled better compared with many natural polymers. Materials such as PCL, PLA, and PLGA can degrade over weeks to months. For this reason, they can provide structural support and long-term drug release. Therefore, synthetic polymers are useful for scaffolds that need to be handled during surgery or used under load. The main limitation of many synthetic polymers is their lack of natural bioactivity. They are generally hydrophobic and do not contain cell-binding sequences. As a result, cell adhesion may be limited unless surface modification is applied. PCL is a common example of a synthetic polymer. It is biocompatible and biodegradable, but its hydrophobic surface usually needs modification [70,71]. Blending with gelatin or other natural polymers, or grafting bioactive peptides, can improve wettability and support cell attachment.

Hybrid Systems: No single polymer class is usually sufficient by itself. Natural polymers provide better biological performance, while synthetic polymers provide better mechanical and degradation control. For this reason, hybrid electrospun systems that combine natural and synthetic components have gained attention. These systems can combine ECM-like bioactivity with mechanical integrity, processability, and controlled degradation, which are needed for reliable and predictable drug delivery [72]. The classification, physical and mechanical properties, main advantages, and biomedical applications of commonly used polymers are summarized in Table 2.

### 4.2. Fiber Morphology and Advanced Architectures

In addition to polymer chemistry, fiber morphology and architecture have an important effect on membrane performance in drug delivery, tissue engineering, and filtration applications. The PHBV-based study shows that processing parameters can change the nanofiber microstructure. Different morphologies can be obtained, including smooth and uniform fibers, bead-on-string structures, porous beads, and hierarchical networks. Each morphology has different functions.

Smooth, Bead-Free Fibers: Under optimized electrospinning conditions, smooth and bead-free fibers can be produced from PHBV solutions. In the reported study, these conditions were about 20 kV applied voltage, 0.5–1 mL/h flow rate, 12 wt.% PHBV concentration, and 0.21 mm needle inner diameter. The fiber diameters were generally around 1–2.5 µm. These fibers had continuous cylindrical morphology, smooth surfaces, and submicron to micron-sized pore networks. This type of structure can provide controlled permeability, mechanical stability, and predictable degradation. For this reason, smooth and bead-free fibers are useful for scaffolds that support cell growth, nutrient transport, and controlled drug delivery [74].

Bead-on-String Morphologies: Different morphologies can be obtained when the electrospinning parameters are not in the optimized range. Lower voltage, higher flow rate, or polymer concentration outside the stable range can cause bead-on-string morphology. In this structure, thin fiber segments and spherical or elliptical beads are observed together. The bead size can be around 4–16 µm. For example, spinning at 9 kV or at a high flow rate of about 5 mL/h can lead to high bead formation and thicker fibers. These beads are mainly formed because of jet instability and incomplete stretching. In many cases, beads are considered defects. However, in drug delivery systems, bead-on-string structures can also act as micro-reservoir architectures. The beads can contain locally enriched drug regions, change the diffusion path along the fiber, and increase surface roughness for better tissue anchorage [74,75].

Porous Beads: At higher flow rates and intermediate voltages, porous beads can be formed in the PHBV system. Fine microcavities are observed on their surfaces. These porous beads can significantly increase the accessible surface area. Therefore, adsorption-based or diffusion-based drug loading can be improved. For this reason, porous beads are attractive for high-capacity or rapid-release systems. They can also be useful for immobilizing biomolecules or catalysts in multifunctional membranes [74].

Parameter Control: Fiber morphology and architecture can be controlled by changing the processing parameters. Higher applied voltage can stretch the jet more and reduce bead formation. Because of this, more uniform fibers can be produced. Low flow rates help the Taylor cone stay stable and support smooth fiber formation. However, high flow rates can cause bead formation and porous structures because excess solution is present and the solvent may not evaporate completely.

Needle inner diameter also affects fiber morphology. A smaller needle diameter can help produce finer fibers with fewer beads. A larger needle diameter can increase droplet formation, clogging, and morphology variation. For PHBV, stable jets and consistent fiber diameters were obtained at 12–14 wt.%. If the polymer concentration is too low or too high, bead formation can occur because of phase separation or insufficient chain entanglement [74].

Advanced Architectures and Their Functions: These morphologies can be arranged as advanced architectures such as smooth fibers, bead-fiber composites, porous beads, and micro-networked webs. These architectures affect drug loading capacity, release kinetics, mechanical performance, barrier properties, and degradation behavior. Hierarchical structures that combine fibers and beads can create multi-scale porosity and high surface areas. This can improve filtration efficiency and increase potential drug-storage volume. Therefore, careful control of electrospinning conditions is needed to design fiber architectures suitable for biomedical and drug delivery applications [74]. This study was included to demonstrate how electrospinning parameters can control fiber morphology, porosity, and surface area. These properties can affect breast cancer drug-delivery systems because they can affect drug loading, release behavior, and tissue interaction.

### 4.3. Effect on Drug Loading, Mechanical Properties, and Degradation

Drug loading, mechanical behavior, and degradation should be considered together in electrospun drug delivery systems. These factors affect how much drug can be loaded, how strong the scaffold is, and how it changes during release. The PCL/PLGA-tenofovir (TFV) system is a good example because it shows how drug incorporation and polymer blending can change electrospun fiber performance [73]. Although tenofovir is not an anticancer drug, this example is included because it demonstrates how drug–polymer interactions can affect release behavior, mechanical stability, and degradation in an implantable electrospun system.

Drug Loading Capacity: Electrospun PCL/PLGA fibers can contain TFV loadings of 10–40 wt.%, which is a significant amount for nanofibers. This loading capacity is related to TFV-polyester interactions in both semi-crystalline PCL and amorphous PLGA domains. These drug–polymer interactions strongly affect fiber behavior. For example, at 15 wt.% TFV loading, the expected increase in Young’s modulus with increasing PLGA content is reduced. This shows that TFV can change the polymer microstructure. Surface aggregates are observed in PCL-rich fibers after stretching, which indicates TFV-rich domains and poor compatibility between TFV and PCL. In contrast, PLGA-rich fibers maintain smoother surfaces, showing stronger interaction between TFV and PLGA. In the clinically used 20PCL/80PLGA blend, the mechanical properties remain stable up to 40 wt.% TFV because the changes in crystallinity and chain mobility balance each other [73,76].

Baseline Mechanical Properties: It has been observed that the baseline mechanical properties of drug-free blends cover a wide range. Pure PCL fibers are soft and ductile with high elongation, while pure PLGA fibers are much stiffer and show necking behavior related to glassy polymers. The modulus increases about five times from 100% PCL to 100% PLGA. TFV incorporation changes the mechanical trends, but it does not significantly change tensile strength. Tensile strength generally remains in the range of 3–6 MPa across compositions [77]. The modulus and strength of the 20/80 PCL/PLGA blend remain stable even at high TFV loading. For this reason, the 20/80 PCL/PLGA blend is suitable for a high-loading drug-eluting scaffold [73].

Fiber Orientation Effects: When fibers are aligned and loaded parallel to the tensile axis, they show higher strength and stiffness. When fibers are randomly arranged in a mat, the mechanical response becomes lower and more distributed. When fibers are loaded perpendicular to the fiber axis, the mechanical performance is reduced. Indeed, fiber orientation can therefore help reduce the mechanical weakening caused by drug loading [78].

Degradation and Mechanical Evolution: Degradation studies indicate that drug release and polymer hydrolysis both affect the mechanical evolution of electrospun fibers over time. In PBS, drug-loaded fibers lose stiffness and strength quickly because TFV release occurs. The Young’s modulus of 20/80 PCL/PLGA fibers can decrease by 60–90% within the first hour. The tensile strength can also decrease by 50–60% within the same period. These changes in stiffness and strength occur before significant polymer hydrolysis occurs. This suggests that drug diffusion alone can weaken the fibers by creating pores and micro-voids. After the initial stiffness loss, degradation and mechanical changes continue as the fibers age. At later time points, such as 240 h, both drug-free and drug-loaded fibers show decreases in performance, with reductions of up to 90% in modulus and 80% in tensile strength. These findings indicate that PLGA hydrolysis is responsible for most of the later mechanical loss. The formation of microcracks also contributes to this loss [73].

Mass Loss and Morphological Changes: Mechanical degradation is accompanied by mass loss, scaffold shrinkage, and morphological changes. Mass loss increases as TFV loading increases. Scaffold shrinkage can increase from about 37% to more than 60% after 240 h when the drug content increases from 15 to 40 wt.%. SEM images show the formation of nodules, pores, and hollow regions. These morphological changes can behave as stress concentrators and can initiate failure when the material is loaded. Mechanical stretching can also change the release behavior. Fibers stretched until failure release TFV faster than unstretched fibers. About 70% of TFV is released from stretched fibers in 96 h, while unstretched fibers need about 240 h to release the same amount. This faster release is related to fiber thinning, higher surface area, and the breakup of drug-rich domains during stretching.

Drug Partitioning: The mechanical model shows that TFV partitioning is not uniform in blended fibers. Approximately 80% of TFV is associated with PCL, while about 20% is associated with PLGA. This uneven distribution explains the change in material behavior over time and the release behavior of TFV. It also shows that phase-specific TFV partitioning should be considered during the design of electrospun polymer systems.

Overall, these findings show that drug loading, mechanical performance, and degradation are closely related. High drug loading can be achieved without major mechanical loss when polymer blends and fiber orientation are optimized. However, drug release and polymer erosion can reshape the fiber structure over time. Therefore, drug loading, polymer blend selection, fiber orientation, release behavior, degradation, and fiber structure should be designed together to keep the scaffold mechanically stable during the whole release period [73].

## 5. Encapsulation of Therapeutic Agents

Electrospun nanofibers support localized drug delivery by allowing different therapeutic agents to be incorporated into these fibers. These agents can include chemotherapeutic drugs, biologics, immunomodulators, and natural anticancer agents. The high surface area and adjustable porosity of electrospun nanofibers help control drug loading and release behavior. Also, because their structure is similar to the extracellular matrix, they can support cell attachment and tissue integration.

Different methods are available for drug loading therapeutic agents into electrospun fibers. These methods include emulsion-based electrospinning, coaxial electrospinning, and post-spinning surface modification. The selected method affects how the drug is distributed inside the fibers and how the drug is released. Polymer selection also affects drug encapsulation, release kinetics, and structural stability.

Polymer-based electrospun systems have been widely investigated for cancer therapy applications. These systems can encapsulate and deliver chemotherapeutics, peptides, proteins, immunomodulators, and combination therapies. Therefore, electrospun nanofibers can provide controlled and sustained release, improve therapeutic efficiency, and reduce systemic toxicity [79,80].

### 5.1. Chemotherapeutics

Electrospun nanofibers are widely used for the localized delivery of chemotherapeutic drugs. In cancer therapy, maintaining a high drug concentration at the tumor site while reducing drug exposure in the rest of the body is the main goal. Previous studies have shown that small-molecule drugs can be incorporated into electrospun polymeric fibers. The distribution of the drug inside the fiber directly affects the release behavior. It has also been reported that polymer type, drug polarity, electrospinning method, and fiber morphology all influence the performance of electrospun nanofibers in chemotherapy applications. These properties support the use of electrospun nanofibers for localized chemotherapy [24,81].

#### 5.1.1. Mechanistic Principles of Chemotherapeutic Encapsulation

In the design of chemotherapeutic-loaded electrospun fibers, drug–polymer compatibility strongly influences drug loading and release behavior. Hydrophobic drugs such as paclitaxel and cisplatin are not highly soluble in water. These drugs can be distributed more uniformly in hydrophobic polymers such as PLA, PLGA, and PCL. As a result, slower drug release can be obtained as the polymer degrades. On the other hand, hydrophilic drugs such as doxorubicin and 5-fluorouracil are more water-soluble and may show poor compatibility with hydrophobic polymers. This can make uniform drug distribution more difficult in these systems. Paclitaxel and cisplatin are examples of drugs that show better compatibility with hydrophobic polymers such as PLA, PLGA, and PCL [81,82].

In many electrospun fibers, drug accumulation occurred near the fiber surface. This would cause an initial burst release, followed by slower release through diffusion or polymer degradation. Kenawy et al. reported that the location of the drug in the fiber has an important effect on the release rate [83].

#### 5.1.2. Strategies to Control Release Profiles

Different electrospinning methods help control drug release. Blend electrospinning is one of the common methods. In this method, the drug and polymer are mixed in the same solution. It is a simple method and can be useful for hydrophobic drugs such as PTX and CIS. These drugs can stay well in hydrophobic polymers such as PLA, PLGA, and PCL. Drug release can occur in stages as the polymer degrades. However, when hydrophilic drugs are mixed with these polymers, high initial burst release can be observed.

Another strategy is coaxial or core–shell electrospinning. In this method, the drug is placed in a protected core and surrounded by a polymer shell. This structure can provide better control of release behavior and is useful for drugs such as DOX and 5-FU. Core–shell systems produced with chitosan/PLA or PVA/PCL have been shown to release drugs over about 25–30 days, with approximately 80–85% total drug release [84].

Emulsion electrospinning is another approach used to obtain core–shell-like structures. In this method, drug- and polymer-containing emulsions are electrospun by using a single nozzle. This method is relatively simple, like blend electrospinning, but it can improve the encapsulation of hydrophilic drugs and provide smoother release profiles.

Drugs can also be loaded after fiber production by post-spinning loading or surface immobilization. This approach can protect sensitive drugs from solvent exposure during electrospinning. However, this method may cause an initial burst release. For this reason, additional layers or surface coatings can be applied to reduce the burst effect. In general, drug–polymer compatibility and hydrophilic/hydrophobic balance are important for release control. Additives such as PEG, gelatin, PVA, and chitosan can also be used to improve the system. These additives can help convert burst-release systems into more controlled and sustained release systems [84].

#### 5.1.3. Representative Chemotherapeutics and Release Behavior

Various chemotherapeutic drugs, including DOX, 5-FU, PTX, and CIS, have been used in electrospun nanofiber systems. DOX is a hydrophilic drug. For this reason, it can be released quickly from simple PLA or PLGA blends. However, when core–shell designs are used, or PEG is added to polyester-based systems, the DOX release can be extended to about 30 days. In these systems, about 80–85% of the drug can be released.

5-FU is also a hydrophilic chemotherapeutic drug. When core–shell systems such as PVA/PCL or chitosan/PLA fibers are used, 5-FU can be released slowly over about 25–30 days. In PLA/gelatin systems, the release can be faster, and about 90% release can be obtained within 10 days. PTX has low water solubility and shows better compatibility with hydrophobic polymers such as PLA and PCL. For this reason, PTX release generally occurs over 30–40 days. When dextran is blended with PLA, a steady release close to 90% can be obtained over about 35 days [85].

CIS is another chemotherapeutic drug that can be incorporated into electrospun fibers. In chitosan/PCL fiber systems, approximately 65% release can be obtained in 30 days. When PCL meshes are modified with additional components, the release period can be extended, and sustained release can be achieved for about 70 days [86].

In general, drugs with low water solubility such as PTX usually show more sustained release behavior. However, hydrophilic chemotherapeutic drugs need specific design strategies to prevent rapid or burst release. Architectural approaches such as core–shell structures, emulsions, and hydrogel additives help control release behavior and improve sustained drug delivery [42].

#### 5.1.4. Therapeutic Efficacy and Translational Challenges

In vitro studies have shown that electrospun nanofibers loaded with chemotherapeutic agents such as doxorubicin (DOX), 5-fluorouracil (5-FU), paclitaxel (PTX), and cisplatin (CIS) can inhibit the proliferation of cancer cells. These systems have been investigated against different cancer types, including breast cancer, lung cancer, glioblastoma, colorectal cancer, and ovarian cancer. In addition to reducing cancer cell growth, chemotherapeutic-loaded nanofibers can also induce apoptosis. This effect is related to changes in apoptosis-related markers, such as decreased BCL-2 expression and increased caspase-3 expression [18].

In vivo studies have shown that drug-loaded electrospun membranes can be implanted locally at tumor sites. With this approach, tumor size can be reduced, and survival outcomes can be improved. In addition, systemic toxicity can be reduced compared with conventional systemic chemotherapy. However, electrospun chemotherapeutic delivery systems have not yet received regulatory approval for widespread clinical use. Future studies should focus on better-controlled materials and fiber architectures that can provide predictable release and safe degradation under clinical conditions [17].

### 5.2. Peptides, T-Cell Therapeutics, and Albumin as Functional Biomaterials

Peptides are important biomolecules because many biological functions in tissues, organs, and cells are regulated by them. They can be produced in various sizes and structures and are generally considered safe for biomedical use. For this reason, peptides have gained attention as therapeutic agents for cancer treatment. Peptides are composed of amino acid sequences and can adopt specific conformations and form stable interactions with molecular targets.

Peptides used in cancer therapy are generally classified into different groups. Cell-penetrating peptides (CPPs) facilitate the intracellular delivery of drugs and other therapeutic agents. Tumor-targeting peptides are designed to recognize and bind to receptors on cancer cells. Therefore, treatment specificity can be improved.

Tumor-targeting peptides can provide different therapeutic effects. For example, membrane-active peptides can disrupt cellular membranes and induce apoptosis in tumor cells. Tumor-targeting peptides have also gained attention because they may help reverse drug resistance in breast cancer cells by regulating drug efflux and intracellular signaling pathways. These peptides can penetrate tissues, show low toxicity, and bind specifically to target molecules. Therefore, they can target cancer cells more selectively and reduce off-target effects [21,87,88].

T-cell immunotherapies have significantly changed cancer treatment. In these therapies, T cells can recognize tumor-associated antigens presented on cancer cells and attack tumor cells. Several strategies have been developed to improve T-cell-based therapies. One method is adoptive cell transfer, where T cells are removed from the tumor, expanded outside the body, and then returned to the patient. Another method is to improve the activity of endogenous T cells by blocking inhibitory signaling pathways. When these inhibitory signals are blocked, T cells can recognize and kill tumor cells more effectively. These approaches have shown strong potential in cancer therapy and show the importance of T cells in tumor control [89].

Electrospinning has also been investigated as a platform to support T-cell-based applications. Electrospun scaffolds have a high surface area and an ECM-like structure, which can mimic the natural cellular microenvironment. Fiber stiffness and structure can be adjusted, and these properties can affect T-cell activation and cytokine production. T-cell mechanobiology is related to how T cells interact with their physical environment and how this interaction affects their behavior. Electrospun systems can support cell attachment, cell growth, and cell collection. Since substrate elasticity and surface chemistry can be controlled, these platforms are promising for large-scale cell expansion and immunotherapy applications [90].

Albumin is an important biomaterial for drug delivery because it is biocompatible and naturally found in blood. Different types of molecules can be carried by albumin, and its clearance is relatively slow. Therefore, it can remain in the body for a longer time. The EPR effect may support the tumor accumulation of systemically administered nanoscale albumin carriers, but it does not explain the behavior of a locally implanted albumin nanofiber. Albumin may also interact with proteins such as gp60 and SPARC, which supports its use as a drug carrier [91].

Albumin-based materials have useful properties for biomedical applications, such as low toxicity and controlled biodegradation. Under suitable conditions, albumin can be processed into fibrous structures similar to native tissue architecture. These fibers can support cell attachment, proliferation, and tissue integration. Their mechanical properties can also be improved to obtain better performance. Previous studies have shown that albumin-based scaffolds are beneficial in wound healing, tissue repair, and cardiac regeneration. Therefore, albumin-based fibers can be considered as a flexible platform for drug delivery and regenerative medicine [92,93,94].

The incorporation of albumin and peptide-based therapeutics into electrospun systems can support localized and sustained drug delivery. In these systems, biocompatible protein-based materials can be combined with synthetic polymers such as PCL or PLGA. This combination can help control therapeutic release while maintaining suitable structural properties.

Electrospun systems can also support local treatment after surgery. For example, they deliver drugs or cells to the tumor removal site. With this strategy, systemic exposure can be reduced, and therapeutic efficiency can be improved. Therefore, electrospun systems may provide a practical platform for localized cancer therapy.

However, incorporating living cells into electrospun fibers remains technically difficult. Applied voltage and solvents can negatively affect cell viability. Recent developments, such as reduced-voltage techniques and improved cell integration methods, have helped to produce more cell-compatible electrospun fibers. These advances may support the development of safer and more effective cell-based or drug-loaded systems for post-surgical cancer treatment [95].

### 5.3. Immunomodulators

Electrospun nanofibers are useful platforms for immunomodulatory therapies. For both tumor suppression and tissue regeneration, the local immune environment should be controlled. Because of their ECM-like structure, electrospun scaffolds can interact closely with immune cells such as neutrophils, macrophages, dendritic cells, and T cells. At the same time, immunoregulatory agents can be delivered locally and in a controlled manner [96]. Some immunomodulatory examples discussed in this section were initially used for nerve or bone regeneration. They are included because they demonstrate how electrospun fibers can influence and affect cytokine delivery, macrophage behavior, and local immune response. These mechanisms are also relevant to the design of local cancer treatments.

#### 5.3.1. Rationale and Mechanisms

Electrospun fibers can function as localized depots for immunomodulator release. These systems can release immunomodulatory agents in spatially and temporally controlled patterns. This allows early control of excessive inflammation, including neutrophil activation and NET formation. In addition, macrophage polarization toward pro-regenerative M2 phenotypes can be promoted. T cells and dendritic cells can also be activated for antitumor immune responses. In long-term disease conditions, local immune balance can also be supported.

In many regenerative and oncological applications, treatment efficiency depends on the transition from an initial inflammatory phase to a pro-resolving and regenerative phase. For this reason, electrospun scaffolds are useful because time-controlled release can be added to the system [21,97].

#### 5.3.2. Classes of Encapsulated Immunomodulators

Cytokines such as IL-10 and IL-4 can be encapsulated directly into electrospun systems or delivered by plasmid-based approaches. Core–shell fibers with two-phase IL-10 release have been developed to reduce fibrosis and support M2c macrophage polarization. In these systems, a small amount of cytokine is released rapidly at the beginning, and then a higher and more sustained release is obtained.

Dual delivery systems have also been studied. For example, IL-4 plasmid-loaded liposomes can be placed on the fiber surface, while NGF can be loaded in the core. This type of system has been shown to improve M2 macrophage polarization and nerve regeneration [98]. Pro-resolving mediators, such as aspirin-triggered resolvin D1, have also been incorporated into grafts. These mediators can reduce inflammation and support endothelialization. Therefore, vascular graft performance can be improved [99].

Small-Molecule Immunomodulators: Small-molecule immunomodulators can also be incorporated into electrospun fibers. For example, Telmisartan-loaded PCL/PVP fibers can shift macrophages from M1 to M2 phenotypes. This change can reduce inflammatory responses and support bone regeneration through pathways such as BMP-2 and Smad [96,100]. In addition, the PAD4 inhibitor Cl-amidine has been incorporated into PDO fibers to inhibit NETosis and reduce neutrophil-related tissue damage [101].

Heparin can be added to the surface of medical fibers or implants. It helps prevent blood clot formation and reduce inflammation. For this reason, healing can be improved, and implant performance can be supported. Small-molecule drugs such as Telmisartan are also important because they can reduce inflammation and support tissue regeneration, including bone regeneration [102].

Gene Delivery Constructs: Gene delivery constructs can also be integrated into electrospun systems. For example, IL-12 plasmid-loaded microspheres can be incorporated into fibers to activate immune cells. When these systems are combined with anti-PD-1 therapy, stronger antitumor immune responses can be obtained [103]. Gene delivery constructs can also be used for regenerative purposes. For example, IL-4 plasmid-loaded liposomes can support macrophage-mediated nerve repair and improve tissue regeneration.

Natural Immunomodulatory Compounds: Natural immunomodulatory compounds can also be used in electrospun fiber systems. Compounds such as thymol and tyrosol can be combined with chitosan and carbon nano-onions to improve biological activity. These systems can provide antibacterial, anti-inflammatory, and wound-healing effects. In addition, amino acid- and maltodextrin-based systems can support immune regulation and reduce inflammation by promoting macrophage polarization toward the M2 phenotype.

Across these systems, electrospun fibers may not only serve as passive carriers, but also act as immune-instructive scaffolds by controlling cellular recruitment, immune phenotype, and cell function [104].

### 5.4. Natural Anticancer Agents

Natural anticancer agents such as polyphenols, flavonoids, terpenoids, and alkaloids can act through different mechanisms. These compounds can induce cancer cell death, inhibit cell proliferation, and reduce metastasis. They can also reduce oxidative stress in the body.

However, the clinical use of natural anticancer agents is limited because many of them have poor solubility, low stability, rapid clearance, and low absorption. For this reason, electrospun nanofibers have been investigated as a useful platform to overcome these limitations. These fibers can protect natural anticancer agents, improve their loading, and provide controlled release [105].

#### 5.4.1. Encapsulation and Stabilization

Natural anticancer compounds such as curcumin, quercetin, resveratrol, catechins, and berberine can be encapsulated into electrospun fibers. This encapsulation helps protect these compounds from light exposure and rapid degradation. In this way, electrospun fibers can act as a reservoir for natural anticancer agents.

The porous structure of electrospun fibers can also help control the release of these compounds. Drug release behavior can be affected by polymer hydrophilicity or hydrophobicity, such as the difference between PCL and PVA blends. Fiber diameter, fiber morphology, and fabrication method also influence the release profile. For example, blend electrospinning and coaxial electrospinning can provide different release behaviors. Depending on the system design, compounds such as curcumin, quercetin, and berberine can be released slowly over one to four weeks [106].

#### 5.4.2. Photo-Catalytically Enhanced Natural-Agent Delivery

A promising strategy is the combination of photocatalytic nanoparticles with natural-agent-loaded electrospun fibers. Nanoparticles such as TiO_2_, ZnO, MoS_2_, Bi_2_O_3_, and AuNPs can generate reactive oxygen species (ROS) under light or near-infrared irradiation. This can improve the anticancer activity of natural agents.

These fiber systems can provide controlled drug release and light-triggered ROS production at the same time. The generated ROS can create oxidative stress in cancer cells and support cell death. Photocatalytic nanoparticles and bioactive natural compounds in the fibers can work together to inhibit tumor growth. For example, curcumin-loaded PCL/ZnO fibers have been shown to generate ROS and kill breast cancer cells under light exposure [107]. These systems can also support light-triggered curcumin release.

Similar fiber systems loaded with resveratrol, quercetin, or berberine have been investigated for glioblastoma, melanoma, and ovarian cancer. These fibers have shown anticancer effects by disrupting mitochondrial function, inhibiting cell proliferation, reducing angiogenesis, and supporting immune response. Therefore, curcumin-loaded PCL/ZnO fibers and similar bioactive systems have important potential for targeted cancer therapy [108,109].

#### 5.4.3. Advantages and Applications

Electrospun delivery systems are effective for carrying hydrophobic natural anticancer agents. The structure and composition of the fibers can be manipulated to better control drug loading and release behavior. This will support localized cancer treatment because therapeutic agents can be delivered to the target area while systemic side effects can be reduced.

Electrospun delivery of natural anticancer agents can also support combination therapy approaches, such as chemotherapy and phototherapy. In these systems, the location and timing of therapeutic activation can be controlled, and these properties may support less invasive tumor treatment and post-surgical recovery [106].

### 5.5. Combination Therapies

Electrospun nanofibers are effective platforms for combination therapy because different therapeutic agents can be delivered within the same system. Their flexible structure allows the incorporation of chemotherapeutic drugs, natural compounds, cytokines, gene therapy agents, and small-molecule immunomodulators. These agents can also be separated spatially and temporally in the fiber structure. This allows electrospun nanofibers to support treatments requiring more than one therapeutic mechanism. Complex diseases such as cancer and chronic wounds involve several biological processes, including inflammation, immune evasion, and uncontrolled cell proliferation. In many cases, a single treatment may not be sufficient. Electrospun fibers can be designed to deliver different types of therapeutic agents at the same time. These agents can include hydrophilic and hydrophobic drugs, cytokines, plasmids, and other bioactive molecules.

The release behavior can also be adjusted according to the treatment need. For example, one agent can be released during the inflammatory phase, another agent can be released during the regenerative phase, and another agent can be released during tissue remodeling. In this way, the therapeutic effect can be localized at the disease site. These systems can also support immune regulation, tumor elimination, and tissue repair. Therefore, electrospun fibers can be used as multifunctional delivery systems for cancer and wound healing applications [110].

#### 5.5.1. Representative Combinations

Some of the following combination-delivery examples were developed for regenerative applications rather than breast cancer. They are included to demonstrate how electrospun fibers can separate different therapeutic agents and provide controlled or sequential release. These design principles may also be useful for combination treatment after breast cancer surgery. Cytokines and growth factors can be combined in electrospun systems to support both immune regulation and tissue repair. For example, IL-4 plasmid-loaded liposomes can be placed on the fiber surface, while NGF can be loaded into the fiber core. This type of dual delivery system can promote M2 macrophage polarization, reduce inflammatory mediator production, and support Schwann cell activity. As a result, nerve regeneration can be improved. These combined systems can provide stronger therapeutic effects than single-agent delivery because different biological pathways are targeted at the same time [111].

Immunotherapy and gene therapy can also be integrated into electrospun platforms. For example, patches containing IL-12 plasmid-loaded microspheres have been developed to activate dendritic cells. When these systems are combined with checkpoint blockade agents such as anti-PD-1, they can produce stronger immune responses. This combination can promote antitumor T-cell responses and improve tumor regression [112].

Small molecule immunomodulators and anti-inflammatory drugs can also be incorporated into electrospun fibers for immuno-regenerative applications. Telmisartan-loaded fibers are one example of this approach. These fibers can reduce inflammatory signaling and support osteogenesis. Therefore, inflammation control and tissue regeneration can be promoted together. These effects show the therapeutic potential of electrospun systems with immunomodulatory properties [96].

Electrospun fibers can also be combined with external stimulation methods such as phototherapy. In these systems, light-responsive components can generate reactive oxygen species (ROS), which can support cancer cell killing and immune activation. At the same time, immunoregulatory agents can be released from the fibers. For this reason, electrospun fibers combined with phototherapy or other external stimuli can provide a multifunctional approach for cancer treatment [96].

#### 5.5.2. Multi-Pathway Targeting and Advantages

Immune response and tissue repair pathways are closely connected. If only one pathway is targeted, such as macrophage polarization or NETosis, the therapeutic efficacy may be limited. For this reason, electrospun fibers may be designed with multiple agents to affect several biological processes at the same time. These processes include neutrophil activation, macrophage behavior, dendritic cell recruitment, and T-cell activation. When these pathways are controlled together, a stronger immune response and better tissue repair can be obtained. This is also important for tumor treatment because the immune system works through many connected mechanisms.

Electrospinning allows different therapeutic components to be integrated into one system. Core–shell, multilayer, or surface-functionalized fibers can separate agents in different regions of the fibers. The timing of release can also be controlled, and biphasic or sequential release profiles can be obtained. In addition, electrospinning can incorporate both small molecules and biologics. Therefore, chemical and immunological components can be combined in one implantable device, which makes electrospun systems useful for combination therapy approaches [104]. Table 3 summarizes the selected published electrospun nanofiber studies related to breast cancer. These studies used breast cancer cell models and/or animal models to examine their responses to the fiber systems and therapeutic agents. The table is not intended to provide an exhaustive list of all published studies.

## 6. Characterization Techniques and Comparative Results Across Studies

### 6.1. Fiber Morphology and Structural Characterization

Understanding the morphology and structure of electrospun nanofibers helps evaluate their mechanical behavior, drug release performance, and biological interaction. In the literature, different characterization methods are used to examine electrospun nanofibers. These methods include scanning electron microscopy (SEM), transmission electron microscopy (TEM), atomic force microscopy (AFM), and three-dimensional imaging techniques.

Primary Imaging Methods: SEM is one of the most commonly used methods for electrospun fiber characterization. It provides high-resolution images and allows the evaluation of fiber diameter, surface morphology, bead formation, and qualitative porosity. SEM is also useful for measuring fiber size from images. However, it mainly provides surface information and cannot fully show the internal structure of the scaffold [133]. TEM is useful for examining small internal details, especially core–shell structures and nanoparticles inside fibers. However, very thin samples are required, and the whole scaffold structure cannot be easily evaluated by this method. AFM can also be used to examine surface morphology at the nanoscale and to measure surface roughness. These surface features can affect protein adsorption and cell adhesion. However, AFM usually examines only small surface areas and is limited for large-scale scaffold evaluation [89].

Three-Dimensional Characterization: Three-dimensional characterization provides information beyond surface morphology. Techniques such as nano-computed tomography (nano-CT) and confocal laser scanning microscopy (CLSM) can be used to examine internal architecture, pore connection, and scaffold organization. Nano-CT can provide high-resolution three-dimensional images and can help evaluate fiber connectivity and pore size distribution [134].

CLSM can also be used to examine hydrated scaffolds and cell–material interactions. This allows electrospun scaffolds to be evaluated under wet conditions, which are closer to the biological environment [135].

Comparative Morphological Features Across Studies: Fiber characteristics vary widely across studies because they are affected by fabrication methods and processing conditions. Conventional needle-based electrospinning generally produces fibers with diameters in the range of 100–800 nm. Needleless or multi-jet electrospinning systems can produce thicker fibers, generally between 500 nm and 2 µm, because fiber control is more difficult in these systems. Coaxial and triaxial electrospinning methods can also produce thicker fibers because more than one fluid is processed at the same time [136].

The collector design also has an important effect on fiber alignment. High-speed rotating collectors can produce highly aligned fibers, while stationary collectors generally produce randomly oriented fiber mats. Fiber packing density and alignment can also affect porosity and pore size. Therefore, key morphological parameters such as fiber diameter, alignment, and porosity are important for scaffold performance.

Fiber morphology can be adjusted by changing electrospinning conditions and processing techniques. The surface pores of electrospun mats are usually in the micrometer range, around 1–10 µm. In contrast, hybrid micro/nanofiber systems can show larger pore sizes, approximately 20–100 µm. Larger pore sizes can improve cell infiltration and tissue integration.

However, no single fabrication method can provide all desired structural properties at the same time. For this reason, a combination of characterization techniques is usually required. Surface imaging methods such as SEM and AFM can be combined with three-dimensional imaging methods such as nano-CT and CLSM. These combined methods provide a more complete evaluation of electrospun nanofiber morphology and architecture [136].

### 6.2. Thermal and Mechanical Characterization

For electrospun scaffolds used in drug delivery and breast cancer-related soft-tissue reconstruction, thermal and mechanical properties should be well understood. These properties are important because they show whether the scaffold is stable, strong, and suitable for use in the body.

Previous studies have shown that differential scanning calorimetry (DSC), thermogravimetric analysis (TGA), and tensile testing are useful methods for scaffold characterization. These tests help to evaluate drug–polymer interactions, polymer degradation behavior, thermal stability, and mechanical performance over time. Therefore, thermal and mechanical characterization helps determine whether electrospun scaffolds are suitable for drug delivery and breast cancer-related tissue reconstruction [137,138,139].

#### 6.2.1. Thermal Behavior: DSC and TGA

DSC is used to analyze polymer crystallization, melting behavior, and glass transition temperature. Drug incorporation can affect these thermal properties. For example, when drug molecules are well dispersed in the polymer matrix, polymer chain packing can be disrupted in electrospun fibers. The absence of characteristic drug peaks can also indicate that the drug is distributed in an amorphous or molecularly dispersed form rather than forming separate drug crystals.

This behavior can improve drug dispersion, which may improve dissolution and support longer drug release. TGA is used to evaluate thermal stability and degradation behavior. Drug-loaded PCL and PLGA systems generally show multi-step degradation behavior, where polymer degradation and drug degradation may occur at different stages. In some cases, drug incorporation can slightly decrease the degradation temperature because the drug can disturb polymer chain organization. However, these systems generally remain thermally stable at physiological temperatures, which supports their use in implantable applications. Additives or crosslinking strategies can also be used to improve thermal stability and long-term scaffold performance [137,138,139].

#### 6.2.2. Mechanical Properties and Implant Performance

Mechanical properties of electrospun scaffolds influence surgical handling, suturing, implantation, and resistance to forces in the body. Electrospun scaffolds are usually flexible because of their fibrous structure, but they may have lower strength than bulk polymer materials. Drug incorporation can also reduce mechanical strength, especially when drug crystals are formed and disturb the fiber structure.

Breast reconstruction studies show how scaffold mechanical properties can affect clinical use. Medical-grade PCL scaffolds implanted for 12 months have been reported to show a modulus of about 1.157 MPa, which is close to the modulus of regenerated tissue, about 1.058 MPa. This indicates that the elastic modulus of PCL scaffolds can be suitable for soft-tissue support [139].

Medical-grade PCL scaffolds can help maintain breast shape without being too stiff. They can also support the regenerating stromal tissue because their structure is maintained over time. Since PCL degrades slowly, gradual tissue remodeling can be supported. For breast reconstruction after cancer surgery, mechanical compatibility helps maintain the implant shape and stability.is important. The implant should be strong enough to prevent deformation, reduce pressure on grafts, and resist collapse during daily movement. Synthetic polymers such as PCL can provide better tear resistance and easier suturing compared with weaker natural fiber-based materials. Therefore, PCL-based scaffolds can provide appropriate mechanical support for implantable breast reconstruction systems [139].

#### 6.2.3. Mechanical Suitability for Breast Cancer Surgical Sites

For breast reconstruction, biomaterial scaffolds should be mechanically strong but also compatible with surrounding soft tissues. The scaffold should have elasticity close to native adipose and glandular breast tissue. In general, tensile values in the range of 0.5–2 MPa are considered relevant for these soft-tissue applications. In addition, the scaffold should tolerate surgical handling, including cutting, stretching, and implantation, without permanent deformation or sagging.

The scaffold should also provide temporary support during tissue regeneration. In many cases, this support is needed for about 6 to 24 months. During this time, degradation should occur in a controlled manner. Controlled degradation can reduce adverse biological responses and allow gradual tissue integration and remodeling.

PCL-based electrospun or manufactured scaffolds are suitable for these applications because they have favorable mechanical properties and biodegradability. These scaffolds can degrade in a controlled way while maintaining enough mechanical support for adipose tissue regeneration. Their mechanical behavior is also close to native breast tissue, which provides a balance between stiffness and flexibility.

Therefore, PCL-based electrospun or fabricated scaffolds may support breast reconstruction after surgical procedures such as lumpectomy or mastectomy. They can also be useful for localized drug delivery applications in post-surgical cancer treatment [139].

### 6.3. Drug Loading and Release Profiles

Electrospun nanofibers are effective drug delivery systems because they have high surface area and can be designed with different fiber architectures. Their flexible structure allows control over how much drug is loaded and how the drug is released. This is important for localized and sustained drug delivery applications.

Drug loading and release behavior are affected by several factors. These include the electrospinning method, polymer type, fiber architecture, and drug–polymer compatibility. Methods such as emulsion electrospinning and coaxial electrospinning can change encapsulation efficiency, burst release, sustained release behavior, and total release duration. Depending on the formulation and encapsulation strategy, drug release can occur over a few hours to several months [37].

#### 6.3.1. Encapsulation Efficiency Across Electrospinning Methods

Encapsulation efficiency is an important factor for therapeutic performance because it shows how much of the active molecule is successfully incorporated into the fibers. Chalco-Sandoval et al. compared emulsion electrospinning and coaxial electrospinning, and this study provides useful information for drug-loaded nanofiber design [140].

In emulsion electrospinning, a hydrophobic or immiscible core phase is mixed with a hydrophilic polymer solution to form an oil-in-water emulsion before electrospinning. The success of this method strongly depends on emulsion stability. Without surfactant, encapsulation efficiency was reported to be about 69%. When a suitable surfactant amount was used, around 0.16–0.32%, encapsulation efficiency increased to about 89–92%. This improvement is related to better droplet stabilization and more uniform distribution in the PVOH solution. However, when the surfactant concentration was too high, above 0.48%, encapsulation efficiency decreased to about 71%. This decrease can occur because micelles are formed and part of the drug is dissolved in the external phase [140].

Emulsion electrospinning can provide high encapsulation efficiency, but it has some limitations. This method is sensitive to moisture. Under humid conditions, fibers can swell and drug leakage can occur. This is especially important for hydrophilic drugs and long-term implantable systems.

Coaxial electrospinning uses two concentric fluid streams to produce core–shell structured fibers. In this structure, the shell layer, which is usually formed from polymers such as PCL, surrounds the core material. The shell acts as a protective layer and helps control the release of the active agent. This architecture allows relatively high drug loading while improving stability and functional performance.

The core–shell structure also limits drug diffusion to the external environment and reduces premature leakage. In addition, these fibers can maintain their structure under high humidity better than conventional fibers. For this reason, coaxial electrospinning is suitable for sensitive therapeutics such as peptides, proteins, and biologics, and sustained release profiles can be obtained.

Emulsion electrospinning is widely used in pharmaceutical applications, especially for hydrophilic drugs. However, burst release and sensitivity to environmental conditions such as moisture can still be observed. In comparison, coaxial electrospinning provides better control over drug release kinetics and better protection of encapsulated agents. Therefore, it can be more suitable for unstable or sensitive compounds.

Triaxial electrospinning is a more advanced approach because multilayered fiber structures and sequential drug release can be achieved. This method allows the incorporation of several therapeutic agents with controlled temporal release profiles. Although triaxial systems are more difficult to optimize, they provide more flexibility and control for advanced drug delivery applications [140].

#### 6.3.2. Burst Release vs. Sustained Release Across Polymer Systems

Burst release and sustained release are important release behaviors in electrospun drug delivery systems. Huang and Brazel explained these release mechanisms in polymer-based drug delivery systems, and this information can also be used to understand electrospun fibers [37].

Burst Release: Burst release occurs when a large amount of drug is released in a short time, usually within minutes to hours. This can happen for several reasons. If the drug is located near or on the fiber surface, it can be released rapidly after contact with the release medium. Drug migration during solvent evaporation can also increase surface drug accumulation. In addition, porous or phase-separated polymer matrices can support faster drug diffusion and cause burst release. Hydrophilic polymers such as PVA, PEO, and HEMA can also increase burst release because they absorb water quickly and swell.

SEM and swelling studies have shown that drug-rich regions near the surface can be responsible for the fast initial release. This is commonly observed in hydrophilic electrospun fibers, where water uptake and swelling accelerate the first release stage [37].

Sustained Release: Sustained release occurs when the drug is released slowly over a longer period. This behavior is mainly controlled by diffusion, swelling, and polymer degradation. Hydrophobic polymers such as PCL, PLA, and PLGA can reduce rapid drug release because they limit water penetration and slow drug diffusion. In more crystalline or dense polymer matrices, drug movement becomes more difficult, which supports longer release. Hydrogel-based materials can also provide sustained release because they swell in water and form a diffusion barrier.

Biodegradable polymers can release drugs in multiple stages. At first, drug diffusion can occur, and later, polymer degradation or erosion can contribute to release. Processing methods such as annealing, crosslinking, and multilayer coating can also reduce burst release and extend the release period [37].

Hydrophilic materials such as PVA and HEMA are widely used in drug delivery systems because they interact well with water. However, in many cases, extra modifications such as crosslinking or increased crystallinity are needed to obtain controlled release behavior. Therefore, these materials are useful for applications that require tunable release profiles.

Due to their high surface area and small fiber diameter, these release behaviors can be more pronounced in electrospun nanofibers. The pore structure of electrospun nanofibers can also be adjusted according to the needs of different applications. These properties make electrospun nanofibers suitable for drug delivery applications [37]. The effect of polymer type on burst release and sustained release behavior is summarized in Table 4.

#### 6.3.3. Reported Release Durations: Hours to Weeks to Months

The review by Gaydhane et al. shows that electrospun fibers can provide different release durations, ranging from immediate release to multi-week delivery. This shows the flexibility of electrospun systems for different drug delivery applications.

Short-Term Release: Short-term release, from seconds to a few hours, is useful for oral-dissolving, buccal, and transdermal delivery applications. Immediate-release fibers can dissolve rapidly and release the drug in a short time. For example, PVP and trolamine fibers can completely dissolve in less than 1 min. Cyclodextrin-based polymer-free fibers can also release the loaded agent within 1 min. Carvedilol-loaded PVP/PEG fibers have been reported to release about 80% of carvedilol in 30 min. Curcumin-gelatin fibers can also provide complete release within 30 min. However, when multilayer coatings are added to curcumin-gelatin fibers, the release time can be extended up to 96 h.

Intermediate Sustained Release: Intermediate sustained release can occur over days to weeks. For example, drug-loaded core–shell fibers can release flurbiprofen over 9 days and vancomycin over 17 days. Solvent-spun PCL fibers can release about half of the loaded drug in 12 days, while melt-spun PCL fibers release less than 10% during the same period. Other core–shell systems, such as acid- and gliadin-based fibers, can release drugs over 7 to more than 14 days depending on shell thickness. These examples show that release duration can be adjusted by changing the polymer system, spinning method, and fiber architecture.

Long-Term Release: Long-term release can continue from weeks to months. Gellan-chitosan gastrointestinal fibers can provide multi-week release under pH transitions. Nanofiber-hydrogel injectable depots can also provide extended delivery, which is useful for cancer and bone-regeneration therapies. Hydrophobic polymer systems such as PCL and PLGA can achieve multi-week to month-scale release because of their slow degradation and limited swelling. The review indicates that hydrophobic electrospun fibers can provide release for several months, which is consistent with reported values of 2–6 months for polyester-based long-acting systems [141].

### 6.4. In Vitro Biological Evaluation

#### 6.4.1. Cytotoxicity Assays (MTT, Live/Dead)

Cytotoxicity evaluation is important to determine the biocompatibility of nanofibrous scaffolds. In the study by Podgórski et al., imaging-based viability staining and metabolic assays were used to evaluate PLLA, PU, and PCL nanofibers. The MTT assay showed cell viability close to 100%, suggesting that toxic leachables were not released from the scaffolds. However, inconsistent MTT results were observed in direct-contact experiments. This was mainly related to the interaction between the nanofibrous structure and insoluble formazan crystals. These crystals could be trapped inside the scaffold pores and cause artificially elevated spectrophotometric readings, especially for PU fibers. For this reason, the use of complementary assays was recommended [142].

Confocal microscopy-based Live/Dead staining provided a more reliable evaluation. This method clearly distinguished viable cells as calcein-positive, but the nonviable cells were ethidium homodimer-positive. All nanofiber types supported high cell viability above 75% after 24 h of culture. In extended culture up to 7 days, strong cell proliferation and minimal cell death were also observed. These results show the good cytocompatibility of scaffolds.

Overall, this study shows that imaging-based Live/Dead assays can provide a more accurate and non-interfering evaluation for porous electrospun materials, although the MTT assay is still a commonly used testing assay [142].

#### 6.4.2. Cell Migration and Invasion Assays

Cell migration and invasion assays are important for understanding metastatic potential and the response of cancer cells to microenvironmental cues. Nelson et al. examined the migration behavior of normal mammary epithelial cells (MCF-10A) and breast cancer cell lines (MCF-7 and MDA-MB-231) with various in vitro methods, including scratch assays, Boyden chambers, and a biomimetic electrospun nanofiber system.

The traditional scratch assay provided limited information about migration. MDA-MB-231 cells closed only about 50% of the wound area after 16 h on flat tissue-culture polystyrene. This result indicates that the observed movement was mostly related to non-directional movement and proliferation rather than true invasion behavior. The Boyden transwell assay showed chemotaxis toward CXCL12. MDA-MB-231 cells exhibited about 29% higher migration compared to MCF-7 and MCF-10A cells. However, this method does not provide real-time tracking and cannot fully show migratory paths or topographical guidance effects, as shown in Figure 8.

The aligned electrospun nanofiber migration assay provided a more physiologically relevant platform by mimicking the aligned collagen fibers found at the tumor–stroma boundary. Migration on randomly oriented fibers did not differ significantly among MCF-10A, MCF-7, and MDA-MB-231 cells. In contrast, aligned fibers increased the total migration distance by 98% for MCF-7 cells and 145% for MDA-MB-231 cells compared with randomly oriented fibers, as shown in Figure 9A. When a CXCL12 gradient was applied to aligned fibers, MCF-7 cells showed a 61% increase in total migration distance, whereas MDA-MB-231 cells exhibited substantially greater directional migration. Under this condition, the net migration distance of MDA-MB-231 cells was 82% greater than that of MCF-7 and MCF-10A cells, while MCF-10A cells showed no significant response to CXCL12. These findings demonstrate that both fiber alignment and chemotactic signaling influence breast cancer cell migration in a cell-type-dependent manner [143].

Overall, aligned nanofiber “cell highways” provide a biomimetic platform for studying cancer cell migration. These systems can show both topography-guided and chemokine-mediated migration. Therefore, they can be useful for studying metastatic progression and screening anti-invasive therapies.

### 6.5. In Vivo Performance

#### 6.5.1. Local Retention and Biodistribution

Efficient tumor targeting and long local retention are important for improving the therapeutic efficiency of nanocarrier systems. Bellat et al. developed a glutathione-modified peptide nanofiber (GSH-NFP) to overcome important in vivo limitations, such as rapid reticuloendothelial system (RES) clearance and poor penetration into the tumor. After intravenous administration in mice with orthotopic triple-negative breast tumors, GSH-NFP showed fast and preferential accumulation in the tumor area. This accumulation was higher than that of the other tested nanofiber analogues.

Whole-body fluorescence imaging showed strong signal accumulation at the tumor site. This result was also supported by ex vivo organ imaging, where the tumor tissue showed higher fluorescence intensity than the liver, spleen, and kidneys. These findings indicate that GSH-NFP can improve tumor targeting and biodistribution.

One important property of GSH-NFP is its enzyme-responsive structural change in the tumor microenvironment. The PEG corona of the nanofiber precursors can be cleaved by tumor-associated proteases, especially cathepsin B. After this cleavage, the nanofiber precursors transform from nanoscale fibers of about 100 nm into micrometer-scale interfibril networks. These larger structures can be physically trapped in the tumor matrix. As a result, prolonged intratumoral retention for more than 3 weeks can be obtained. This was confirmed by longitudinal fluorescence imaging and super-resolution STED microscopy.

PET/CT studies also showed progressive and sustained tumor uptake. Radioactivity reached its highest level around day 7 and then slowly decreased over time. Overall, these findings show that GSH-NFP can provide deep tumor penetration, enzyme-induced aggregation, and slow clearance. Therefore, a long-lasting intratumoral depot can be formed, which can increase drug exposure and improve therapeutic outcomes [144].

#### 6.5.2. Tumor Regression and Recurrence Prevention

The nanofiber platform was also evaluated for tumor regression and recurrence prevention by using an aldox-NFP formulation. This formulation combines the tumor-targeting ability of GSH-NFP with a chemotherapy agent that can be activated under specific tumor-related conditions.

In a triple-negative breast cancer model, aldox-NFP was administered at a low dose of 2.5 mg/kg once per week. This treatment reduced tumor size more effectively than free aldoxorubicin and the liposomal formulation Doxil. When the dose was increased to 5 mg/kg, a greater reduction in tumor size was obtained. In a metastatic lung cancer model using MDA-MB-231 cells, aldox-NFP also showed strong inhibition of cancer spread. The longest survival was observed in the aldox-NFP group, with a median survival time of 72 days. This was higher than the survival observed with free aldoxorubicin or Doxil. Mass spectrometry results also showed that aldox-NFP delivered higher levels of doxorubicin metabolites into tumor tissues than the other treatments. This indicates that aldox-NFP can improve drug delivery to the tumor site and maintain the drug in the tumor for a longer time [144].

Aldox-NFP was administered multiple times and did not cause strong systemic toxicity. The liver, kidney, and heart tissues remained normal, and no important abnormalities were observed in blood test results. In contrast, animals treated with Doxil or free aldoxorubicin showed tissue damage, including muscle degeneration and splenic hemosiderin deposition. These results show that the nanofiber platform can provide two important advantages: improved therapeutic efficiency and reduced off-target toxicity. Therefore, aldox-NFP can be considered a promising approach for tumor regression, recurrence prevention, and long-term cancer treatment outcomes [144].

#### 6.5.3. Comparative Summary of Best-Performing Systems

Bellat et al. [144] compared different peptide-based nanofiber precursor (NFP) designs to determine which structural features provide better in vivo performance. Among the tested systems, GSH-NFP showed the best biodistribution, tumor retention, and therapeutic efficiency. Therefore, it was considered the most advanced and best-performing formulation.

Tumor Targeting and Biodistribution: GSH-NFP showed the highest tumor-specific accumulation among the tested analogues with different surface charges and electron-donor modifications. Its optimized zeta potential of about −10 mV supported tumor homing and reduced accumulation in RES organs. Ex vivo fluorescence imaging also showed the highest tumor-to-liver signal ratio for GSH-NFP among all candidates.

Penetration and Enzyme-Induced Local Retention: GSH-NFP showed improved penetration into deep tumor tissue. It also formed strong interfibril networks after protease activation in the tumor microenvironment. This enzyme-induced structural change provided local retention for more than three weeks. In contrast, the non-activatable D-configured analogue, d-GSH-NFP, did not form these structures and showed lower retention. This result shows the importance of enzyme responsiveness.

Pharmacokinetic Stability and Distribution: Among the tested nanofibers, GSH-NFP had the longest circulation half-life, approximately 22 h. It also showed the highest tumor %ID/g value, with a maximum of 13%ID/g. This value was higher than that of typical PEGylated liposomes. These pharmacokinetic properties helped maintain higher drug levels in the tumor and improved therapeutic efficiency.

Drug Delivery Efficiency and Therapeutic Effect: Aldox-NFP showed better performance than free aldoxorubicin and Doxil in both primary and metastatic breast cancer models. Enhanced tumor suppression, delayed metastasis, increased survival, and higher active drug levels in tumor tissues were observed with this system.

Safety and Toxicity Profile: GSH-NFP also showed a better safety profile with minimal systemic toxicity in different organs. This was different from Doxil or free drug treatments, where tissue damage and off-target toxicity were reported. Overall, these results show that GSH-NFP and aldox-NFP are promising systems for tumor targeting, prolonged local retention, improved therapeutic efficiency, and reduced systemic toxicity [140].

## 7. Applications for Breast Cancer Therapy

### 7.1. Rationale for Local Nanofiber Delivery

Breast cancer treatment still commonly includes systemic chemotherapy. However, systemic chemotherapy has several limitations that can reduce treatment efficiency. When chemotherapeutic drugs are delivered through the whole body, healthy tissues can also be damaged. For this reason, side effects such as cardiotoxicity, neurotoxicity, and general weakness can occur [145].

Another limitation is the poor delivery of chemotherapy to the tumor site. Tumor blood vessels are abnormal, and this can reduce drug penetration into the tumor tissue. In addition, multidrug resistance can remove chemotherapeutic drugs from cancer cells and decrease intracellular drug concentration. Therefore, the therapeutic effect can be reduced. These problems show the need for drug delivery systems that can deliver chemotherapy more directly to the tumor region instead of exposing the whole body to the drug.

Electrospun nanofibers can be used as a local drug delivery platform to overcome these limitations. These fibers can release therapeutic agents directly at or near the tumor site. In this way, local drug concentration can be increased, while systemic drug exposure can be reduced. For this reason, electrospun nanofibers have gained attention as promising systems for localized breast cancer therapy [145].

### 7.2. Subtype-Specific Design Considerations for Breast Cancer

Breast cancer subtype should be considered when developing a drug delivery system. Nanofibers can deliver endocrine drugs such as Tamoxifen for ER-positive breast cancer. These drugs may also be combined with other anticancer agents. Suárez et al. [118] studied tamoxifen and curcumin in electrospun fibers using MCF-7 breast cancer cells. For HER2-positive breast cancer, HER2-targeted drugs or antibodies may be useful. However, direct studies using electrospun systems for this subtype are still limited. For triple-negative breast cancer, chemotherapy, immunotherapy, and combination treatments may be more suitable because ER, PR, and HER2 targets are absent. Several studies reviewed in this manuscript used MDA-MB-231 or 4T1 models for aggressive or triple-negative breast cancer [110,113,114,122,125,126,127,129]. Therefore, the drug, release profile, and nanofiber design should be selected based on the breast cancer subtype.

### 7.3. Post-Surgical Implantation Strategies

Surgical removal of the tumor is one of the main treatment regimens for breast cancer therapy. However, residual malignant cells can remain at the resection margins after surgery, and this can cause local recurrence. These remaining cells may not be completely eliminated by systemic chemotherapy because of poor local drug penetration and dose-limiting toxicity [123]. Electrospun nanofibers are suitable for post-surgical implantation because they are flexible, have a high surface-area-to-volume ratio, and contain interconnected porous structures. These properties allow the nanofiber mats to be placed into the surgical area and provide local drug release near the remaining cancer cells. This supports their use as implantable drug delivery systems for post-surgical breast cancer treatment [146,147].

### 7.4. Prevention of Local Recurrence

Breast-conserving surgery can still be associated with local recurrence. This can occur when microscopic residual cancer cells remain in the tumor bed after surgery and start to grow again. Electrospun nanofibers can act as local drug reservoirs at the tumor site. This allows therapeutic agents to be maintained near the tumor bed for a longer period of time instead of being rapidly cleared from the body.

Previous studies have shown that poor drug penetration and rapid systemic clearance can reduce the effectiveness of chemotherapy [145]. Local implantation of drug-loaded nanofibers can help maintain higher drug levels at the tumor site. Studies such as those reported by Liu et al. and Kaplan et al. have shown that drug-loaded nanofibers placed directly at the tumor site can reduce recurrence risk and extend recurrence-free time by providing sustained local drug release [148].

### 7.5. Comparison with Systemic Therapies

Systemic therapies are used to control cancer progression, but they still have important limitations. One major limitation is that only a limited amount of the administered drug reaches the tumor tissue. At the same time, healthy tissues can also be exposed to the drug, which can cause systemic side effects. As reported by Minchinton and Tannock, poor drug penetration into solid tumors is an important barrier for effective cancer treatment [145].

In contrast, electrospun nanofibers can provide controlled local drug delivery directly in or near the tumor bed. This localized approach can reduce systemic exposure while maintaining higher therapeutic drug concentration at the target region. Nanofiber-based delivery systems have been shown to provide better tumor control and reduced side effects in breast cancer models compared with conventional systemic administration [81].

## 8. Tumor Microenvironment Barriers

### 8.1. Hypoxia

Hypoxia is commonly observed in the breast cancer tumor microenvironment. This condition occurs because tumor cells grow rapidly, but the tumor vasculature is not sufficient and is often abnormal. When hypoxia occurs, hypoxia-inducible factors (HIFs) become stabilized. These factors can make breast cancer cells more aggressive and can support treatment resistance, immune escape, and metastasis.

Tumor blood vessels are usually abnormal and do not function properly. In addition, interstitial fluid pressure in the tumor tissue is often high. These conditions can prevent therapeutic agents from reaching the central regions of the tumor. Therefore, systemic therapies may show limited efficiency because the drug cannot penetrate deeply into the tumor core. Hypoxia can also activate resistance-related pathways and reduce the effectiveness of chemotherapy. In radiotherapy, low oxygen levels can reduce treatment response because oxygen is needed for radiation-induced damage. For this reason, hypoxia is considered an important barrier to breast cancer treatment. Abnormal tumor vasculature and high interstitial pressure can limit drug transport to the tumor core, as noted by Minchinton and Tannock [141].

### 8.2. Dense ECM and Diffusion Limitations

Breast tumors usually contain a dense extracellular matrix (ECM) composed of collagen and other structural proteins. This dense ECM can increase tissue pressure and limit drug diffusion. As a result, therapeutic agents may not reach all regions of the tumor equally. In many cases, the outer part of the tumor receives more drug exposure, while the inner regions remain less treated.

This dense matrix can act as a physical barrier against drug transport. Therefore, delivery systems that can improve drug penetration through the ECM are needed. For breast cancer therapy, this is important because the treatment should reach not only the tumor surface but also deeper tumor regions [149,150,151].

### 8.3. Heterogeneous EPR Effect

The enhanced permeability and retention (EPR) effect can vary between tumors and between patients. This variation is mainly related to differences in tumor blood flow, vascular permeability, and tissue structure around the tumor. Because of this heterogeneity, nanoparticles do not always accumulate in tumors in a predictable way.

This creates an important limitation for nanoparticle-based targeting strategies. In breast cancer therapy, the EPR effect may not always provide reliable tumor accumulation. Therefore, delivery systems that do not depend only on passive accumulation through the EPR effect are needed [152].

### 8.4. Nanofiber Adaptations to Overcome TME Barriers

Local implantation can reduce dependence on abnormal tumor vasculature and the heterogeneous EPR effect by placing the drug reservoir near residual tumor cells. However, local placement alone does not guarantee drug penetration through a dense extracellular matrix. Fiber porosity, tissue contact, degradation rate, and drug-release duration should therefore be optimized. In addition, pH- or ROS-responsive fiber designs may be used to respond to acidic and oxidative conditions in the tumor microenvironment. These strategies still require validation in post-surgical breast cancer models [149].

## 9. Stimuli-Responsive Electrospun Nanofibers

Stimuli-responsive electrospun nanofibers allow drug release at the target region and at the required time. These systems can release drugs by responding to internal tumor-related signals or external stimuli. In this way, treatment can become more precise, and drug exposure in healthy tissues can be reduced. Therefore, stimuli-responsive electrospun nanofibers are important for reducing systemic toxicity, which is one of the main limitations of conventional cancer treatments [153].

### 9.1. pH-Responsive Nanofibers

Tumor tissues have a different microenvironment compared with normal tissues. One important difference is the acidic pH of the tumor region. pH-responsive nanofibers can remain stable under normal physiological conditions, but they can release drugs when they are exposed to the acidic tumor environment. The application of certain pH-responsive polymer systems has been made, as they are able to recognize the differentiating aspects of aerated and deoxygenated environments and the capacity of pH-responsive materials for regulated drug delivery systems [38]. This release behavior can increase drug accumulation in tumor tissue. At the same time, drug exposure to other parts of the body can be reduced. For this reason, pH-responsive nanofibers are useful for tumor-targeted drug delivery.

These kinds of nanofibers may contain polymers such as chitosan, poly(acrylic acid), poly(vinyl alcohol) (PVA), and pH-sensitive methacrylate copolymers. For example, coaxial electrospun nanofibers that use PCL as the shell and PVA as the core have been used to provide sustained release of doxorubicin. Yan E’s study showed that at acidic pH (4), degradation is higher, leading to greater drug release compared to normal pH conditions [154].

### 9.2. ROS-Responsive Nanofibers

Tumors usually contain high levels of reactive oxygen species (ROS). ROS-responsive nanofibers can use this condition for controlled drug release. In these systems, oxidation-sensitive linkers can break down in the tumor environment and trigger drug release. This mechanism can improve tumor specificity and may also help overcome resistance-related pathways. Therefore, ROS-responsive nanofibers are promising systems for more selective cancer therapy because these nanofibers contain oxidation-cleavable groups such as thioketal, thioether, boronic ester, or diselenide linkages. Having higher ROS levels around the tumor, in contrast to normal tissue, makes these nanofibers capable of releasing their therapeutic load preferentially in the tumor environment while reducing exposure to healthy cells. For example, Yao et al. demonstrated that electrospun polyurethane fibers contained ROS-cleavable thioketal bonds. Exposure to hydrogen peroxide caused cleavage of the thioketal groups, polymer degradation, and ultimately release of methylprednisolone [155].

### 9.3. Thermal-Responsive Nanofibers

Thermal-responsive nanofibers can be used for drug delivery under mild hyperthermia, which is often used in cancer therapy. When heat is applied, thermosensitive polymers can undergo physicochemical changes. These changes can control the timing and amount of drug release. In this way, treatment control can be improved, and drug release can be adjusted according to the local temperature conditions.

For example, Poly(N-isopropylacrylamide) (PNIPAAm) is one of the polymers that show thermoresponsive behavior due to changes in hydration and chain conformation with temperature. Electrospun PNIPAAm/poly(2-acrylamido-2-methylpropanesulfonic acid) fibers demonstrated the release of nifedipine in response to temperature changes, affecting drug release behavior [156].

### 9.4. Light-Triggered Nanofibers

Light-triggered nanofibers can be activated by light, especially near-infrared light. In these systems, photothermal agents are incorporated into the fibers. When near-infrared light is applied, these agents convert light into heat. This heat can trigger drug diffusion or drug release at the tumor site. Therefore, light-responsive nanofibers can provide localized drug release and improve targeted cancer treatment.

Fibers with this capability contain photothermal compounds, including polydopamine, gold nanorods, graphene oxide, carbon nanotubes, black phosphorus, or indocyanine green. A study by Cen et al. tried to produce electrospun PCL/gelatin fibers with deposited doxorubicin-loaded polydopamine nanoparticles. Polydopamine converts light to heat, accelerating doxorubicin release [157].

### 9.5. Multi-Stimuli Strategies

Multi-stimuli-responsive nanofibers can respond to more than one trigger, such as pH, redox conditions, temperature, and light. These systems are useful because tumors can show different microenvironmental conditions. By responding to multiple signals, drug release can be controlled more effectively. Therefore, multi-stimuli-responsive nanofibers can improve treatment efficiency while reducing side effects [153].

For example, electrospun PNIPAAm–N-methylolacrylamide–acrylic acid fibers reacted to both temperature and pH. They were also able to control the release of curcumin [158]. Moreover, PCL/gelatin electrospun fibers loaded with polydopamine affected the release of doxorubicin in response to pH and infrared radiation [157].

## 10. Limitations of Electrospinning and Drug Formulation

Electrospinning is a valuable method for localized drug delivery, but it has several limitations. The electrospinning process is very sensitive to different formulation, processing, and environmental conditions. Polymer concentration, viscosity, conductivity, solvent properties, applied voltage, flow rate, needle-to-collector distance, temperature, and humidity can affect fiber diameter, morphology, porosity, and reproducibility [41,96]. For this reason, these parameters should be controlled carefully. This becomes more difficult during scale-up, where it is harder to keep the same fiber properties in each batch [159].

Another limitation is the use of organic solvents. Solution electrospinning often requires volatile organic solvents. Some of these solvents can be toxic and may remain in the fibers after fabrication [160]. Residual solvents can reduce biocompatibility and may also change drug release or cell response [160]. Electrospinning can affect sensitive molecules, including proteins, peptides, enzymes, and nucleic acids. Organic solvents, high voltage, shear forces, and interfacial stress are factors reducing stability and biological activity [110,161]. Coaxial and emulsion electrospinning can reduce direct exposure of these molecules to harsh solvents. However, these methods are more difficult to prepare and control [110,162].

Drug–polymer compatibility is also an important challenge. Drugs and polymers can have different hydrophilicity, polarity, and solubility. Because of this, the drug may not be distributed evenly inside the fibers. Phase separation, crystallization, or movement of the drug toward the fiber surface can also occur. When more drug is located near the surface, an initial burst release may happen [18,163]. Hydrophilic drugs can be difficult to combine with hydrophobic polymers such as PCL, PLA, and PLGA. In contrast, poorly water-soluble hydrophobic drugs may need suitable organic or mixed-solvent systems, or other formulation methods [18,163]. Therefore, drug–polymer compatibility and drug dispersion should be checked before electrospinning.

Burst release is another common limitation of drug-loaded electrospun fibers. When drug molecules are located on or close to the fibers’ surface, burst release occurs; this is because when the fibers contact water or biological fluids, these drug molecules can be released quickly. Fiber porosity, drug distribution, polymer hydrophilicity, and phase separation can also affect burst release [37]. In some cases, a fast initial release can be useful. However, uncontrolled burst release can shorten the treatment period and may increase local toxicity. To improve release control, strategies such as core–shell fibers, multilayer structures, crosslinking, and surface coatings can be employed and play a critical role in this regard.

Advanced electrospinning methods also have their own limitations. Coaxial electrospinning requires suitable core and shell solutions. Viscosity, flow rate, solvent compatibility, and solvent evaporation should be carefully controlled. If these conditions are not well matched, unstable jets, defective fibers, or loss of the core–shell structure can occur [33,39]. Triaxial electrospinning can provide better control of sequential drug release. However, it uses several fluid streams and has a more complex setup. This makes optimization and scale-up more difficult. Emulsion electrospinning also depends strongly on emulsion stability. Droplet coalescence or phase separation can cause uneven drug loading and burst release. Surfactants may also create additional biocompatibility concerns [164].

Melt electrospinning does not use organic solvents, which is an advantage. However, it requires high processing temperatures. Because of this, it may not be suitable for heat-sensitive drugs, proteins, peptides, or other biological molecules. In addition, polymer melts usually have high viscosity and low conductivity. Therefore, the fibers produced by melt electrospinning are often larger than those produced by solution electrospinning [165]. Needleless electrospinning can increase the production rate. However, it is more difficult to control jet formation, fiber uniformity, and complex fiber structures [166].

Drug loading can also change the physical and mechanical properties of electrospun fibers, affecting polymer chain arrangement, crystallinity, tensile behavior, fiber morphology, and degradation. Chou and Woodrow showed that drug incorporation and drug release changed the mechanical behavior of PCL/PLGA electrospun fibers [73]. This shows that drug loading, polymer composition, degradation, and mechanical properties should be evaluated together. A higher drug loading does not always contribute to showing better performance. In some cases, high drug loading can weaken the fibers or create drug-rich regions that change the release behavior.

Sterilization is another challenge for drug-loaded electrospun systems. Gamma irradiation, UV treatment, plasma treatment, ethylene oxide, and other sterilization methods can change polymer properties and drug stability. They may also affect wettability, degradation, mechanical behavior, and drug release. These effects depend on both the polymer and the loaded therapeutic agent. For this reason, sterilization should not be evaluated only by checking sterility. Fiber morphology, mechanical properties, degradation, drug content, drug activity, and release behavior should also be tested after sterilization [167].

Overall, electrospun drug-delivery systems need careful optimization. Electrospinning parameters, drug–polymer compatibility, drug stability, loading efficiency, release behavior, mechanical properties, polymer degradation, sterilization, reproducibility, and scale-up should all be considered together. These limitations are still important barriers to the clinical use of electrospun anticancer implants [159,168].

## 11. Translational and Manufacturing Challenges

Electrospun nanofiber drug delivery systems have shown promising results in laboratory studies. However, their translation to clinical use is still limited by several challenges. These challenges include solvent toxicity, biomolecule instability, batch-to-batch variability, sterilization problems, polymer degradation-related tissue responses, and mechanical fixation difficulties. In addition, some nanofiber implants may require surgical placement, which can make clinical use more complex.

### 11.1. Solvent Toxicity

According to ICH Q3C guidelines, many of these solvents are classified as Class 2 solvents, so their residual amount should be kept very low. For this reason, drying steps after electrospinning are important. After fabrication, residual solvent content should also be evaluated. In addition, greener solvent systems are being investigated to improve the safety and sustainability of the electrospinning process [169,170].

### 11.2. Biomolecule Stability

To protect the stability and biological activity of biomolecules, protective electrospinning can be used. For example, coaxial electrospinning and emulsion electrospinning can encapsulate biomolecules inside aqueous droplets or core regions. This helps protect sensitive molecules from direct contact with harsh solvents and electric fields [171]. However, process optimization is still needed. Parameters should be carefully adjusted to reduce interfacial damage and maintain biomolecule function during and after electrospinning [172].

### 11.3. Batch Reproducibility and Quality Control

Electrospinning is sensitive to solution properties, environmental conditions, and processing parameters. Because of this sensitivity, it can be difficult to obtain the same fiber properties in each batch, especially during scale-up. Changes in fiber diameter, porosity, morphology, or drug loading can affect drug release behavior and regulatory acceptance.

For this reason, quality control is necessary for electrospun drug delivery systems. These controls may include SEM analysis for fiber morphology, drug loading measurements, mechanical testing, and in-process monitoring systems. Such controls are needed to improve reproducibility and to meet good manufacturing practice (GMP) requirements [173].

### 11.4. Sterilization Compatibility

Sterilization may alter both the electrospun polymer and the incorporated therapeutic agent. Gamma irradiation can cause polymer-chain degradation and reduce molecular weight. In PCL- and PLCL-based nanofibers, the extent of this change has been shown to depend on the irradiation dose, polymer composition, and initial molecular weight, with greater changes observed at higher doses. Ethylene oxide sterilization avoids high temperatures but may leave residues and can also alter molecular-weight distribution. Autoclaving is generally unsuitable for many electrospun systems because heat and moisture may deform the fibers or destabilize the incorporated drug. Plasma treatment, electron-beam irradiation, and aseptic manufacturing may provide alternatives, but their effects are formulation-dependent. Therefore, sterilization should be evaluated by examining fiber morphology, polymer molecular weight, mechanical properties, degradation, drug content and activity, and release behavior rather than by assessing sterility alone [169,174].

### 11.5. Polymer Degradation and Tissue Response

Polymer degradation behavior strongly affects tissue response. For example, PLGA degradation can produce acidic products, which may cause local inflammation. On the other hand, slowly degrading polymers such as PCL can remain in the body for a long time and may cause a foreign-body response.

To reduce these problems, polymer blending, copolymer design, or buffering additives can help control degradation behavior and reduce inflammation. The polymer degradation rate should match the required treatment period. In this way, the scaffold can provide support and drug release for the needed time while reducing adverse tissue responses [175,176].

### 11.6. Mechanical Fixation Challenges

Mechanical fixation can be a challenge for electrospun nanofiber mats. These mats can be thin and fragile, and they may tear during suturing. Adhesives can be used for fixation, but their long-term stability may be limited. New strategies include direct deposition of nanofibers onto tissue, attachment of nanofiber mats to a backing layer, and use of surface coatings to improve fixation.

Proper fixation enhances the nanofiber mat in contact with the target tissue. If the mat moves or detaches, local drug delivery may become uneven. Therefore, fixation strategies should be designed carefully to maintain tissue contact and provide uniform drug release [177,178].

## 12. Toward Clinical Translation

### 12.1. GMP-Compliant Scale-Up

Clinical translation of electrospun nanofiber systems requires GMP-compliant electrospinning. GMP production helps control solvent handling, process conditions, product reproducibility, and quality. Industrial-scale electrospinning systems can use enclosed chambers for solvent recovery, process monitoring, and product consistency. These systems help reduce batch-to-batch differences and support regulatory requirements. Previous studies have shown that GMP-based production of electrospun products is technically feasible, and this supports the scale-up potential of electrospun nanofiber manufacturing [173,179].

### 12.2. Regulatory Pathways for Combination Products

Drug-loaded electrospun nanofibers are generally considered combination products because they include both a drug component and a device component. For this reason, they should be evaluated in terms of both drug safety and device performance. The regulatory pathway can change depending on the main function of the system.

For approval, detailed information is needed about material safety, drug release behavior, biocompatibility, degradation, and therapeutic efficiency.

In order to translate these methods into devices, several specific tests are often performed: biocompatibility and endotoxin testing, as well as testing of residual solvents and identification of extractables and leachables. More importantly, drug content and release behavior must be confirmed as reproducible across batches. These can be done by local and systemic pharmacokinetic studies. These tests are important as the electrospun drug-containing implants have both a drug aspect and a device aspect. Drug-loaded electrospun nanofibers should also follow Good Manufacturing Practice requirements and medical device quality standards. Therefore, collaboration between material scientists, pharmaceutical researchers, clinicians, and regulatory experts is important for clinical translation [180].

### 12.3. Scalable Production Technologies

Electrospinning has been shown to be a feasible technique for producing nanofibers at a larger scale. Different production technologies, such as multi-needle, needleless, and centrifugal electrospinning, can be used to increase production from laboratory-scale amounts to larger quantities.

Needleless systems are especially useful because they can provide a high fiber production rate and reduce needle clogging problems. However, the production rate and fiber quality should be balanced. Previous studies have shown that fibers produced at larger scales can have similar morphology and drug loading capacity compared with fibers produced at a laboratory scale. When processing parameters are properly controlled, drug release behavior can also remain reproducible. Therefore, electrospinning is a promising method for large-scale nanofiber production [173].

### 12.4. Integration with Personalized and Model-Driven Cancer Therapy

Electrospun nanofiber systems can be adjusted according to tumor-specific requirements. Polymer composition, drug loading, fiber architecture, and release kinetics can be modified for different treatment needs. For this reason, these systems are suitable for personalized cancer therapy.

Computational modeling and machine learning methods are increasingly used to predict drug release behavior and optimize formulation parameters. These approaches can support the design of data-driven and patient-specific implants. Therefore, model-driven design can improve treatment accuracy and help develop more effective localized cancer therapies [24].

### 12.5. Prospects for Clinical Trials

Electrospun nanofiber systems are moving closer to clinical testing. Early studies on electrospun products have shown that these systems can be manufactured and evaluated for safety, which provides a basis for future cancer-related clinical applications. In the first clinical trials, safety, degradation behavior, local tissue response, and localized drug release should be evaluated.

After safety is confirmed, later studies can focus on therapeutic efficiency, such as tumor control and reduction in local recurrence. If these results are successful, electrospun nanofibers may become useful systems for localized cancer treatment. Their ability to deliver drugs locally, reduce systemic exposure, and support controlled release gives them strong potential for future cancer therapy [179]. Currently, electrospun nanofibers are being studied in both oncology and non-oncology clinical trials. In terms of oncology, an ongoing study is evaluating the Restrata^®^ synthetic electrospun fiber matrix to treat surgical defects following the resection of malignant cutaneous neoplasms (NCT06578650). This clinical trial focuses on assessing postoperative wound granulation and healing, not anticancer efficacy [181]. Restrata^®^ is also being evaluated in non-oncologic clinical studies; it is being evaluated for diabetic foot ulcers (NCT04918784), and it achieved complete wound closure after 12 weeks in 74% of the per-protocol patients treated with the electrospun matrix, compared to patients treated with standard care, who showed 33% improvement. These studies suggest that electrospun products can be manufactured, implanted, and clinically assessed. However, anticancer electrospun nanofibers are still at the preclinical stage [182,183].

## 13. Future Directions

Future studies are focused on improving electrospun nanofiber systems to make them more precise, adaptable, and clinically useful. The main goal is to develop nanofiber systems that can provide better patient outcomes. For this reason, smart materials, sensing ability, data-guided design, and advanced scaffold architectures are becoming important research directions.

### 13.1. Smart and Multifunctional Fibers

Stimuli-responsive nanofibers have been investigated in many studies. These fibers can respond to changes in the surrounding environment, such as pH, temperature, light, or magnetic field. When these changes occur, the fibers can release therapeutic agents on demand.

Multifunctional nanofibers can also combine more than one therapeutic function. For example, chemotherapy delivery, immune regulation, and anti-inflammatory effects can be included in the same system. The combination of stimuli-responsive behavior and multifunctional design can be useful for tumors with heterogeneous structure and resistance mechanisms. Therefore, these systems are promising for improving cancer treatment efficiency [25].

### 13.2. Integrated Sensing and Theranostic Fibers

Theranostic nanofibers can combine drug delivery with imaging or sensing functions. These systems can help monitor drug release, tumor response, and local biological changes at the same time. This information can support treatment planning and help clinicians make better decisions during therapy. For this reason, theranostic nanofibers are considered promising systems for personalized and responsive cancer treatment [184].

### 13.3. Machine-Learning-Guided Design

Machine learning can be used to improve the design of electrospun nanofibers. It can help predict fiber morphology, drug loading capacity, and release behavior based on processing parameters and material properties. This data-guided approach can improve reproducibility and reduce trial-and-error experiments. Therefore, machine-learning-guided design can support the development of more reliable and effective therapeutic nanofiber systems [185].

### 13.4. 3D and Hierarchical Architectures

Three-dimensional and hierarchical nanofiber scaffolds can better mimic the structure of natural tissues. These scaffolds can support deeper cell infiltration and provide more controlled drug release. When electrospinning is combined with 3D printing or other fabrication methods, patient-specific implants can be produced.

These advanced architectures can provide better control of local treatment after surgery. For this reason, 3D and hierarchical nanofiber scaffolds are promising for post-surgical cancer therapy and tissue regeneration applications [26].

## 14. Conclusions

Electrospun nanofiber technology is a promising platform for improving cancer therapy by increasing local drug accumulation and providing controlled release profiles. These systems can improve therapeutic efficiency while reducing off-target toxicity compared with conventional systemic chemotherapy. Fabrication techniques such as blend electrospinning, coaxial electrospinning, and other advanced electrospinning methods can also provide better control over drug distribution inside the fiber matrix.

Electrospun nanofibers have been shown to provide effective local drug delivery with reduced damage to healthy tissues in physicochemical, biological, and preclinical studies. In addition, recent developments in multifunctional and theragnostic fiber systems can support more accurate and personalized cancer therapies.

However, several challenges should still be addressed before wide clinical use can be achieved. These challenges include reproducible large-scale manufacturing, long-term safety validation, regulatory approval, and clinical testing in patients. If these problems are successfully solved, electrospun nanofiber technology may become an important part of next-generation cancer therapy by providing more effective and less toxic treatment strategies.

## Figures and Tables

**Figure 1:**
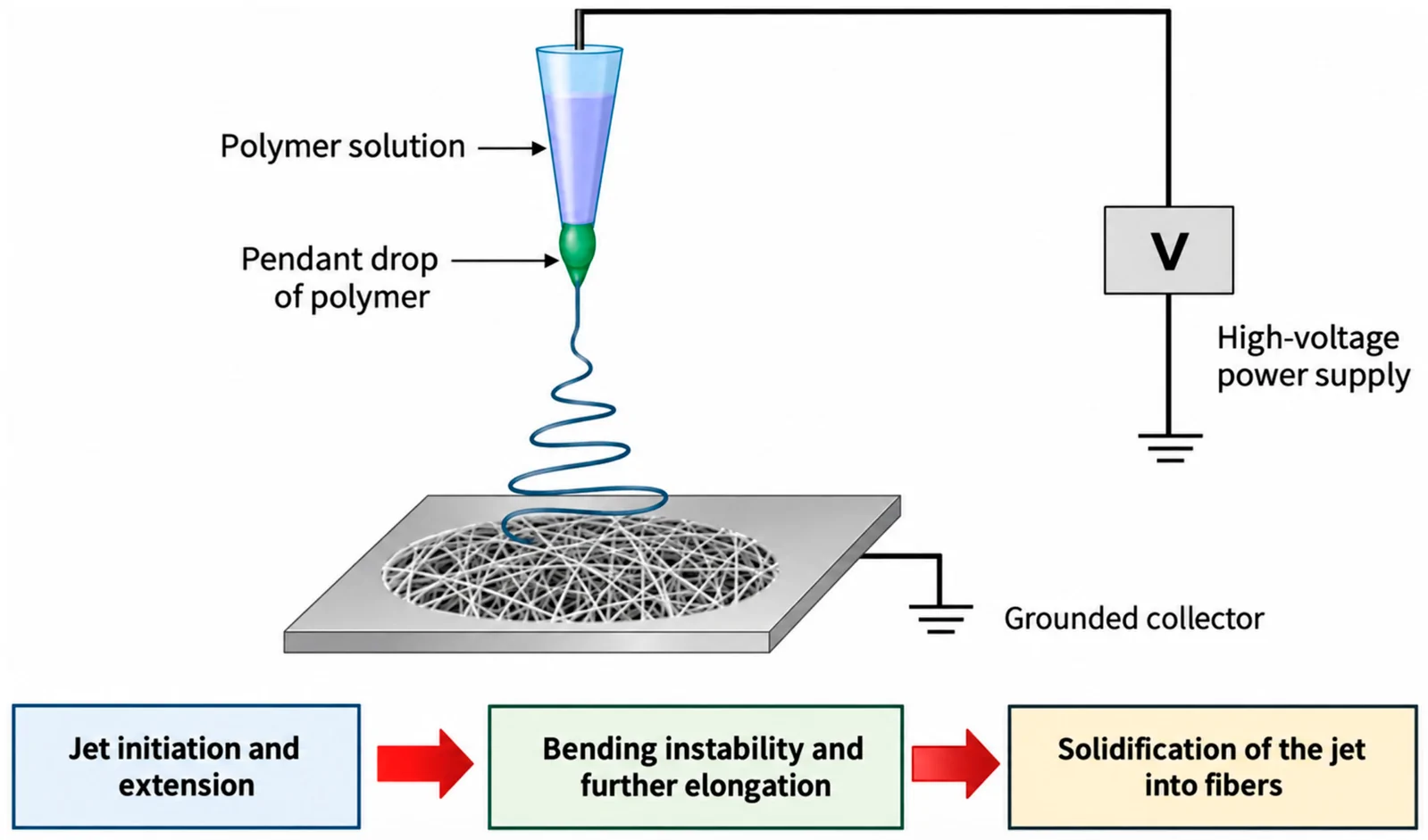
Basic principle of electrospinning. Adapted with permission from Ref. [28].

**Figure 2:**
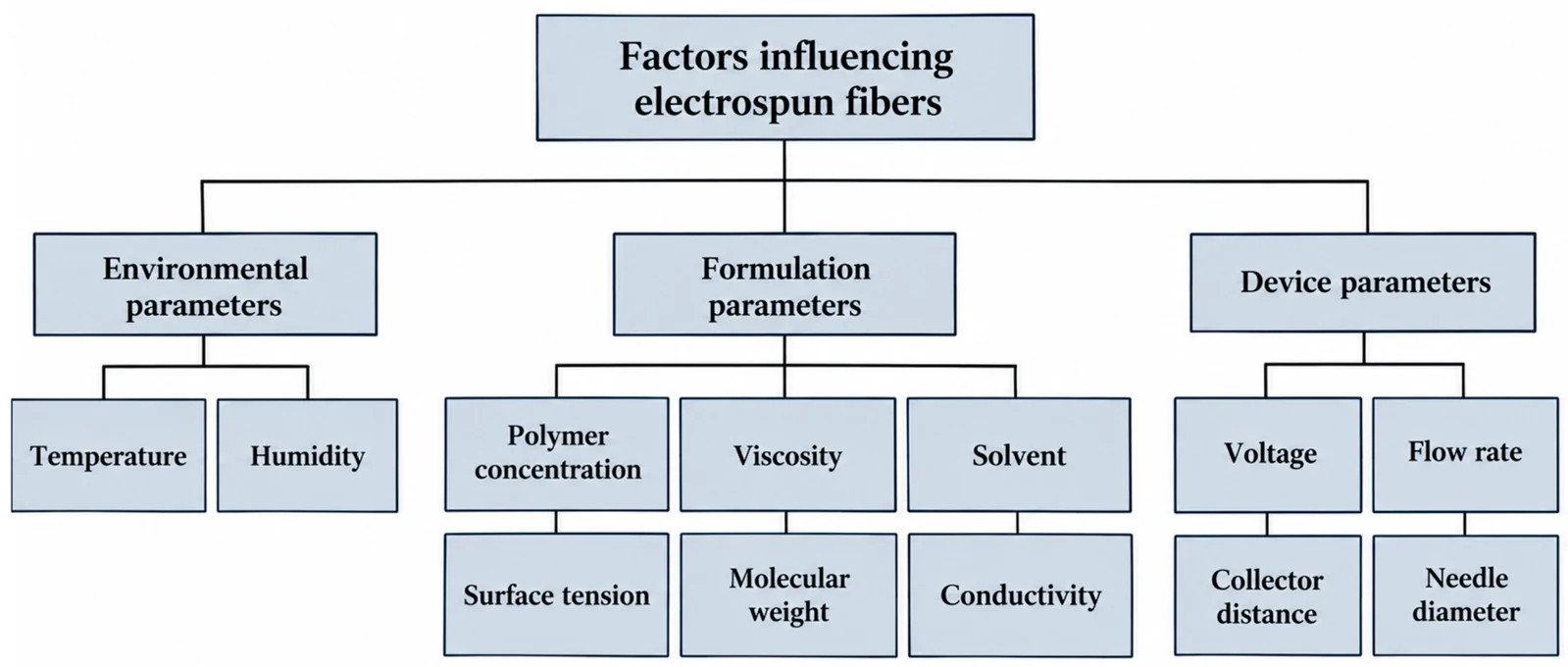
Key electrospinning parameters, including environmental, formulation, and device-related factors. Adapted with permission from Ref. [29].

**Figure 3:**
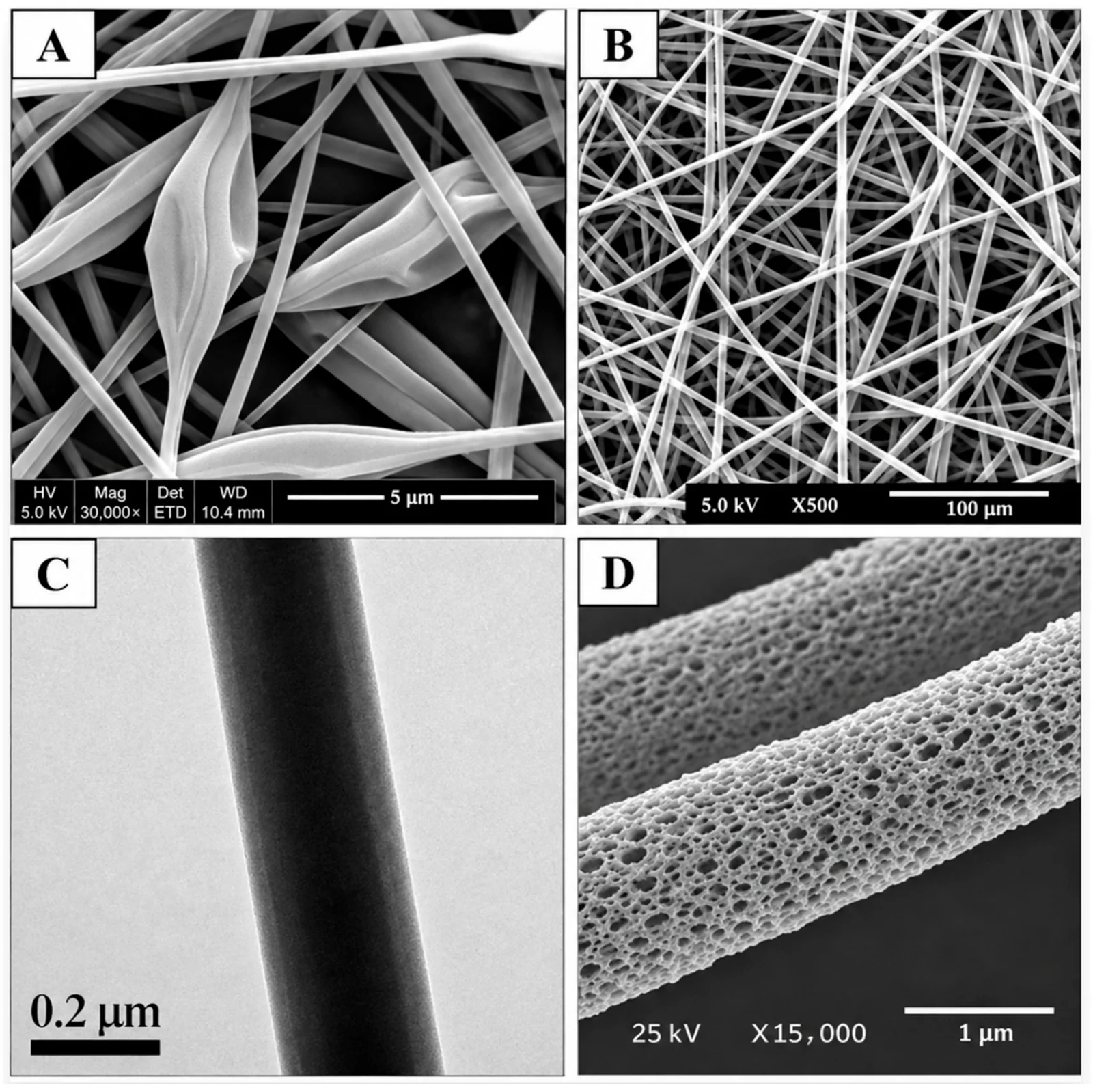
Different fiber morphologies: (**A**) beaded; (**B**) smooth; (**C**) core–shell; and (**D**) porous fibers. Adapted with permission from Ref. [28].

**Figure 4:**
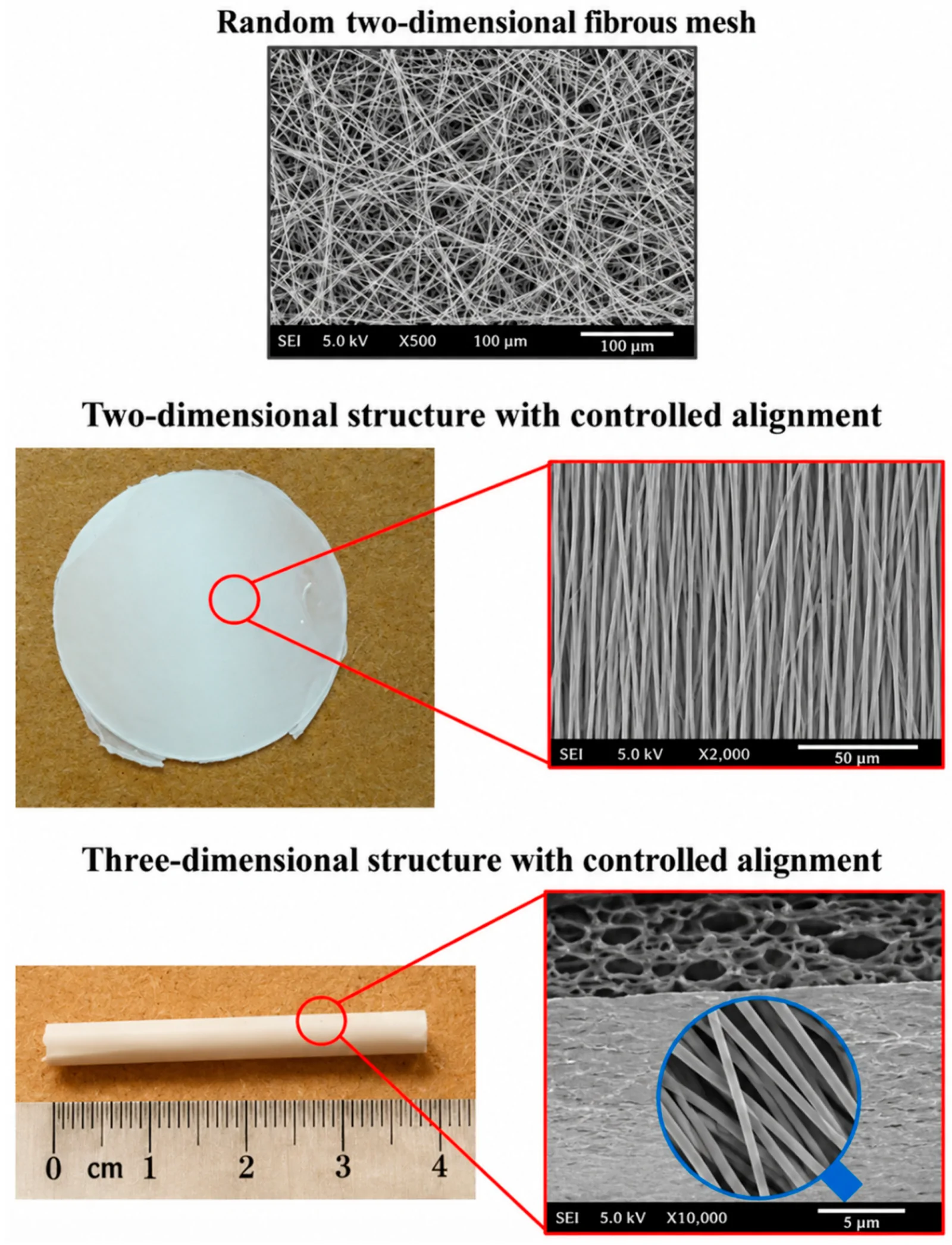
Two- and three-dimensional structures made of electrospun fibers. Adapted with permission from Ref. [28].

**Figure 5:**
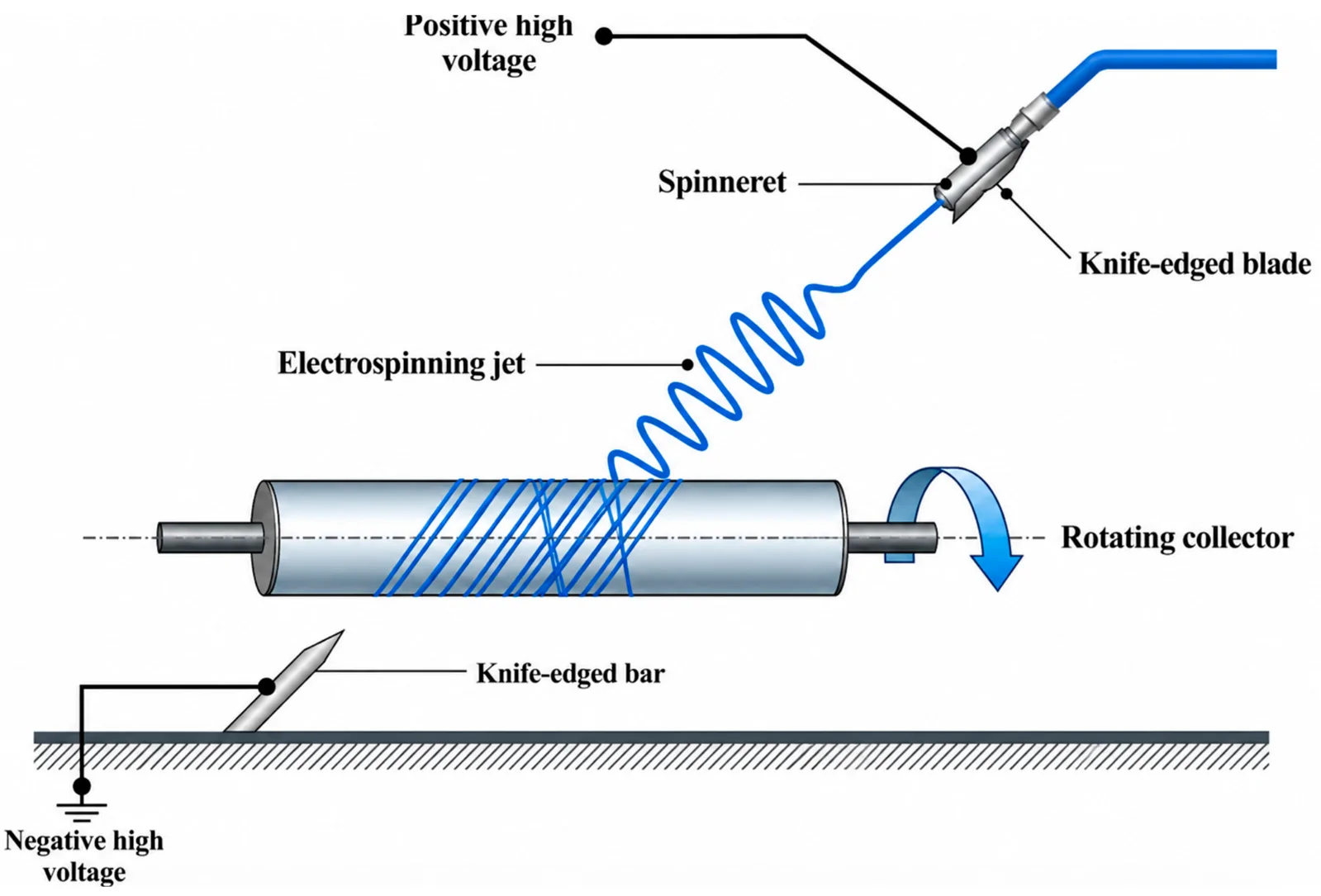
Controlling fiber alignment on a tubular scaffold through mechanical rotation and modification of the electric field. Adapted with permission from Ref. [28].

**Figure 6:**
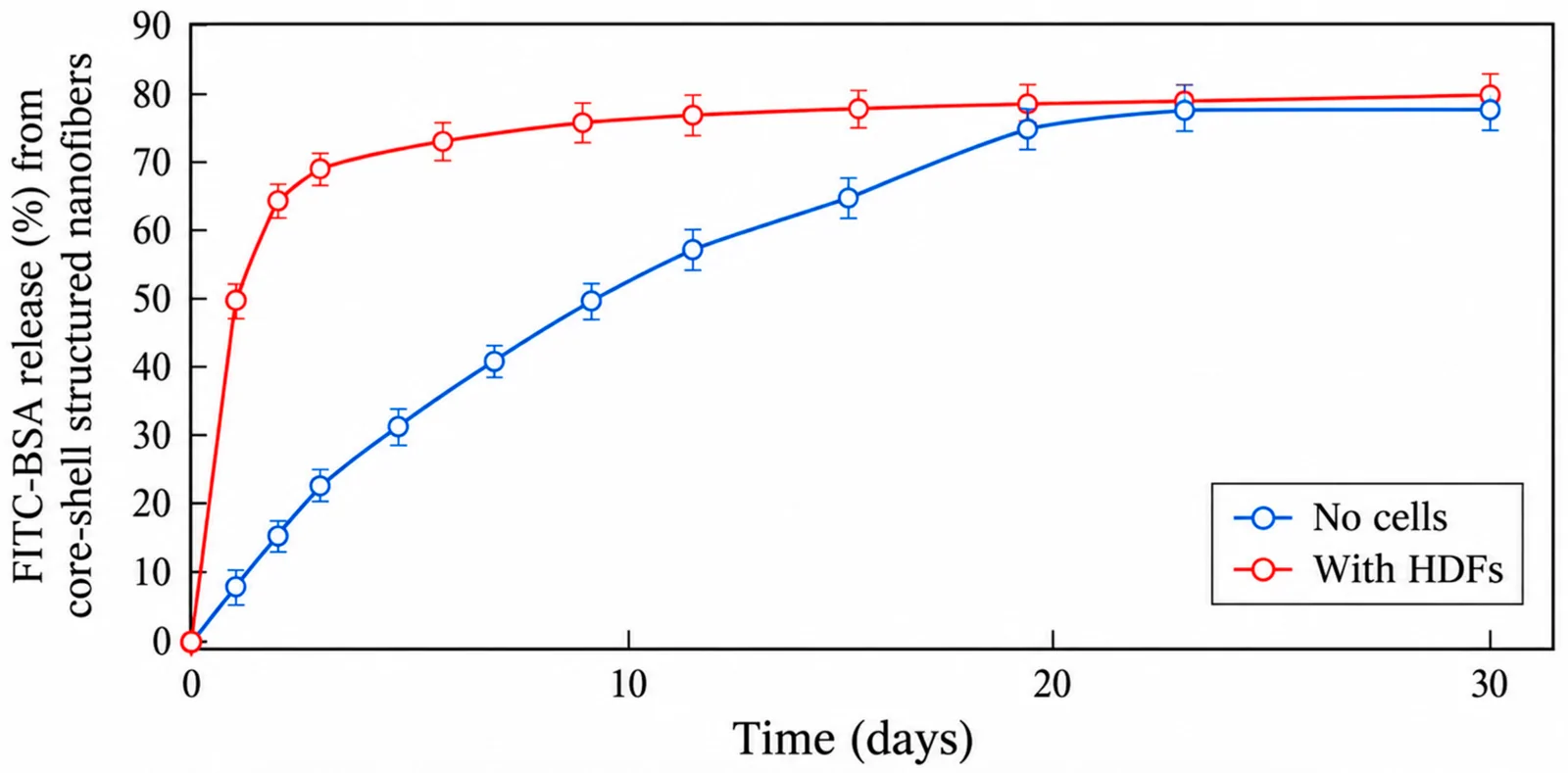
Percentage release of FITC-BSA from PCL core–shell structured nanofibers over time. Faster release was observed for nanofibers cultured with HDFs. Adapted with permission from Ref. [28].

**Figure 7:**
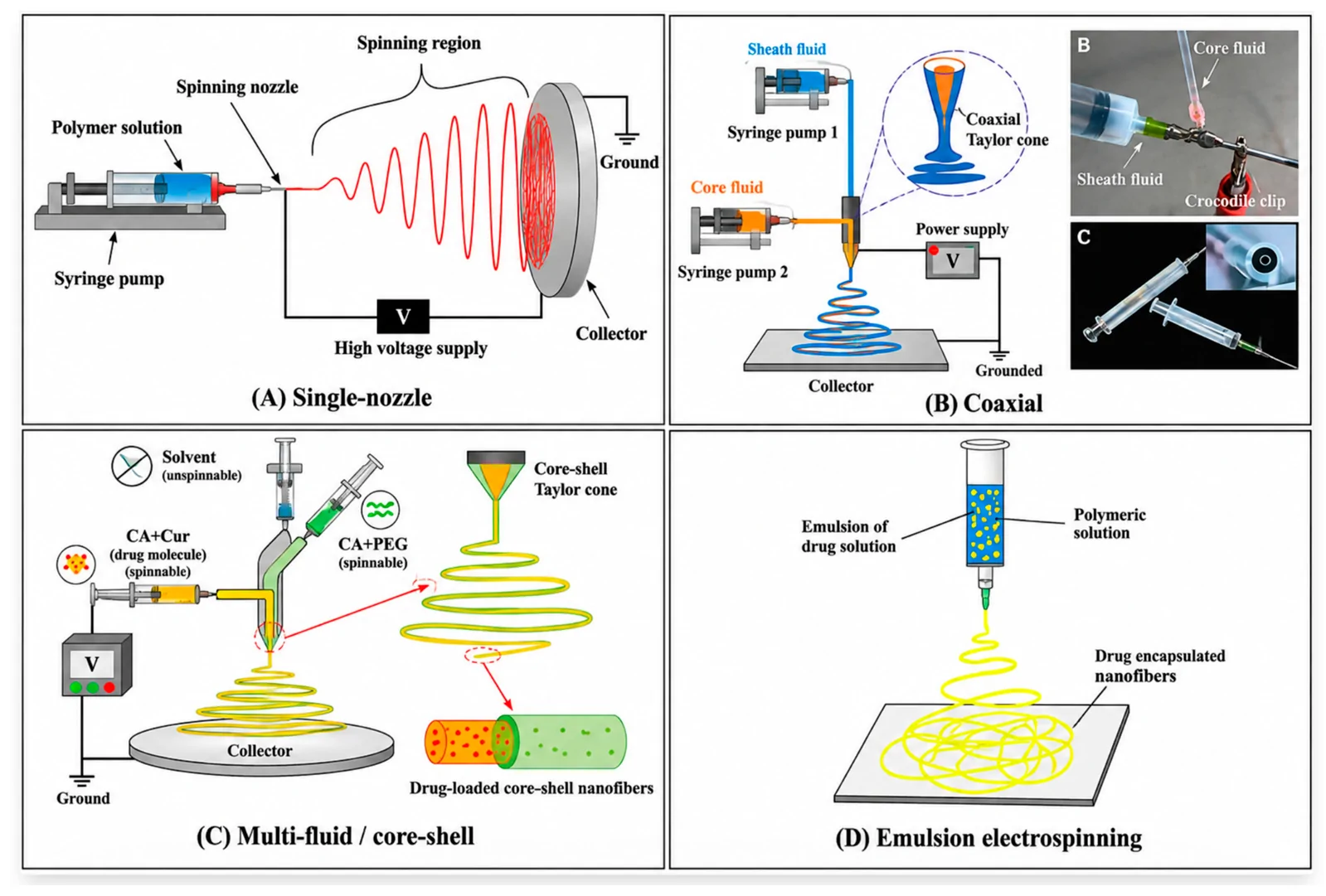
Representative electrospinning configurations used for biomedical nanofiber fabrication: (**A**) single-nozzle electrospinning, Adapted with permission from Ref. [61]. (**B**) coaxial electrospinning, Adapted with permission from Ref. [62]. (**C**) triaxial electrospinning, Adapted with permission from Ref. [63]. (**D**) emulsion electrospinning. Adapted with permission from Ref. [64].

**Figure 8:**
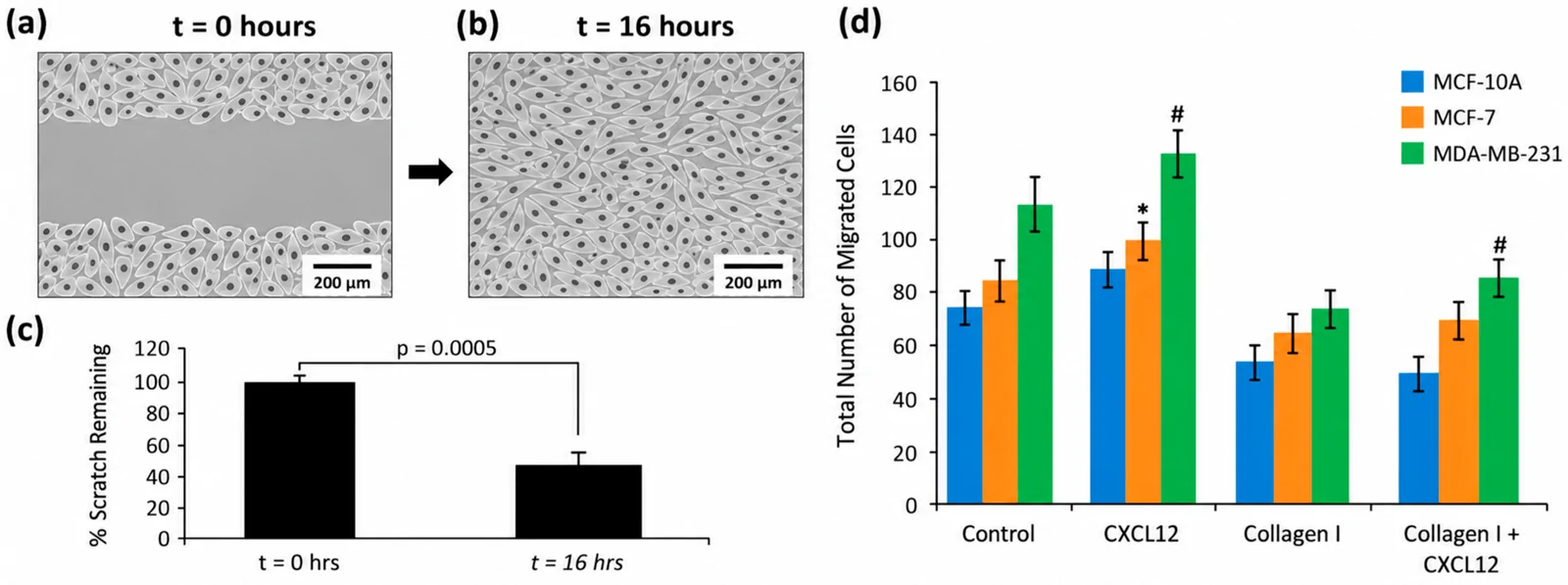
Conventional in vitro assays used to evaluate breast cancer cell migration. (**a**–**c**) Scratch/wound healing assay showing partial closure of the wound area by MDA-MB-231 cells after 16 h on tissue-culture polystyrene, demonstrating limited directional migration assessment. (**d**) Boyden transwell assay illustrating CXCL12-mediated chemotactic migration, where MDA-MB-231 cells exhibited greater migratory activity than MCF-7 and MCF-10A cells. * *p* < 0.05, # *p* < 0.01. Adapted with permission from Ref. [143].

**Figure 9:**
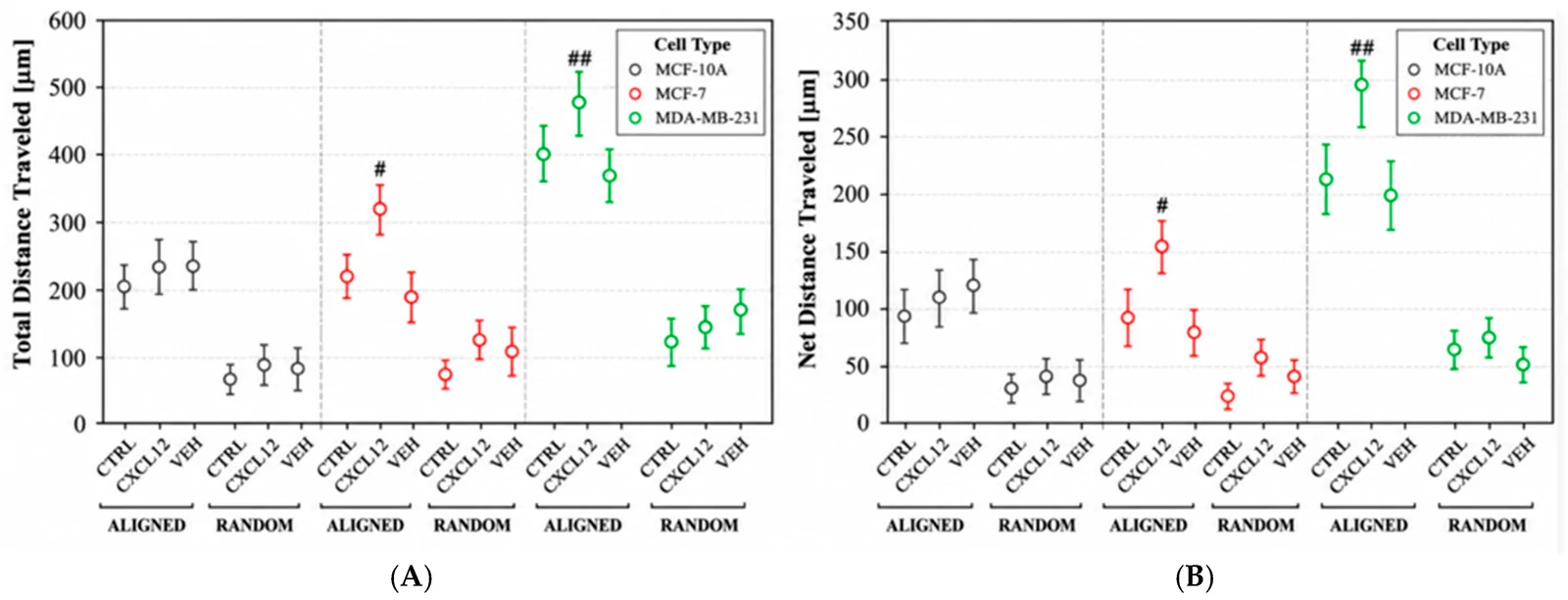
(**A**) Total migration distance of MCF-10A, MCF-7, and MDA-MB-231 cells on aligned and random electrospun nanofibers under control, vehicle, and CXCL12 conditions. (**B**) Net migration distance demonstrating enhanced directed migration in response to CXCL12 gradients, particularly in MDA-MB-231 cells. (# *p* < 0.05, ## *p* < 0.01) Adapted with permission from Ref. [143].

**Table 1:** Comparison of Electrospinning Techniques and Their Potential Relevance to Localized Cancer Therapy.

Technique	Key Features	Principal Advantages	Limitations	Representative Drug/Cargo and Relevant Property	Potential Relevance to Localized Therapy
Single-nozzle [36,40,55]	Simple one-needle setup	Simple operation; broad material compatibility	Uncontrolled or burst release	Paclitaxel (PTX) poorly water-soluble, hydrophobic anticancer drug	Local delivery of small-molecule anticancer drugs
Coaxial [36,41,42,56]	Concentric dual-fluid spinneret; core–shell fibers	Protects proteins and peptides; sustained release	Complex process optimization; release depends on shell composition, thickness, and processing parameters	Doxorubicin hydrochloride (DOX)—water-soluble anticancer drug	Protected delivery of proteins, peptides, and sensitive anticancer agents
Triaxial [36,43,57]	Triple-layer fibers	Multi-stage and sequential release	More complex setup and optimization	5-FU, DOX, and PTX—drugs with different solubility properties; suitable for sequential and sustained combination release	Sequential or combination delivery of therapeutic agents
Melt [45,46,47,58]	Solvent-free polymer melt	No solvent residue, controllable fiber formation	High processing temperature may degrade the polymer and limit the loading of heat-sensitive agents	Daunorubicin hydrochloride—anticancer drug; slow and sustained release	Implantable local-delivery scaffolds when the drug tolerates processing heat
Emulsion [48,49,51,59]	Water-in-oil or oil-in-water emulsion, single nozzle	Protects hydrophilic drugs; simple setup	Emulsion instability, surfactant issues	Hydroxycamptothecin (HCPT)—poorly water-soluble anticancer drug	Delivery of proteins, peptides, and other water-soluble therapeutic agents
Needleless [52,53,54,60]	Free-surface multi-jet	High throughput; multiple simultaneous jets	More difficult control of jet initiation and fiber uniformity	Paclitaxel (PTX)—poorly water-soluble, hydrophobic anticancer drug; sustained local release	Scalable production of drug-loaded fibrous mats

**Table 2:** Classification, physical and mechanical properties, advantages, and biomedical applications of commonly used polymers in electrospun nanofibers.

Polymer	Classification	Main Physical Properties	Mechanical Properties	Main Advantages	Main Applications
PCL [69,70]	Synthetic, biodegradable polyester	Hydrophobic; slow degradation; semicrystalline	Good flexibility and mechanical stability	Easy electrospinning; long-term structural support; sustained drug release	Drug delivery, implantable scaffolds, tissue engineering
PLA [69]	Synthetic, biodegradable polyester	Hydrophobic; relatively slow degradation; semicrystalline	Relatively stiff; good tensile strength	Good processability; biodegradable; suitable for controlled drug release	Drug delivery, tissue engineering, implantable systems
PLGA [69,73]	Synthetic, biodegradable copolymer	Hydrophobic; degradation rate can be adjusted by lactide:glycolide ratio	Moderate mechanical strength; decreases during degradation	Tunable degradation and drug release; widely used in biomedical systems	Controlled drug delivery, cancer therapy, implantable systems
PU [69]	Synthetic polymer	Can be designed with different hydrophilicity and elasticity	High elasticity and good mechanical strength	Flexible; durable; suitable for soft-tissue applications	Soft-tissue scaffolds, wound-related applications, drug delivery
Nylon-6 [69]	Synthetic polyamide	Relatively hydrophilic compared with polyesters; good thermal stability	High tensile strength and toughness	Strong fibers; good fiber-forming ability	Tissue engineering and mechanically stable scaffolds
Gelatin [66,68]	Natural, protein-based polymer	Hydrophilic; water-sensitive; biodegradable	Low mechanical stability in wet conditions	Good biocompatibility; ECM-like properties; supports cell attachment	Tissue engineering, wound healing, drug delivery
Collagen [69]	Natural, protein-based polymer	Hydrophilic; biodegradable; ECM component	Low mechanical strength when used alone	Excellent bioactivity and cell interaction	Tissue engineering, wound healing, ECM-mimicking scaffolds
Chitosan [67]	Natural polysaccharide	Hydrophilic; biodegradable; positively charged	Low-to-moderate strength; may require blending or crosslinking	Biocompatible; antimicrobial; supports cell interaction	Drug delivery, wound healing, tissue engineering
Silk fibroin [65]	Natural protein	Biodegradable; relatively stable in aqueous conditions	Good tensile strength compared with many natural polymers	Good biocompatibility and mechanical performance	Tissue engineering, drug delivery, implantable scaffolds
Cellulose derivatives [65]	Natural-derived polysaccharides	Generally hydrophilic; properties depend on chemical modification	Moderate mechanical stability	Good biocompatibility; adjustable surface properties	Drug delivery, wound dressings, tissue engineering
Hybrid natural/synthetic systems [72]	Composite/blended polymers	Properties depend on polymer combination	Better mechanical balance than many natural polymers alone	Combine bioactivity of natural polymers with strength and processability of synthetic polymers	Drug-loaded scaffolds, tissue engineering, localized therapeutic implants

**Table 3:** Representative electrospun nanofiber systems for biomedical and tissue engineering applications for breast cancers.

Reference	Electrospun Polymer/Platform	Drug/Active Agent	Reported Release Profile	Breast Cancer Model	Main Outcome	Key Limitation
Sadeghi et al., 2023 [113]	Trilayer tubular PLA/PCL and Alg-DA/PVA scaffold	Docetaxel (DTX) + photothermal treatment	N/A; DTX was used during treatment rather than as a fiber-controlled release system	MDA-MB-231	The 3D tubular scaffold supported breast cancer cell growth and enabled evaluation of combined chemo-photothermal therapy, which produced enhanced tumor-cell inhibition.	In vitro model only; no in vivo validation and no scaffold-mediated DTX release profile.
Sedky et al., 2023 [114]	PCL electrospun nanofibers	Nedaplatin + Peganum harmala alkaloid-rich fraction	Initial release followed by sustained release for 8 days	MCF-7; MCF-10A as non-tumorigenic control	Dual-loaded fibers showed the highest antiproliferative activity against MCF-7 cells (IC_50_ = 3.21 µg/mL) and high cancer-cell selectivity.	In vitro evaluation only; postoperative recurrence prevention has not been validated in vivo.
Rabionet et al., 2017 [115]	PCL electrospun scaffold	None	N/A	MDA-MB-231	Provided a 3D breast-cancer culture environment and promoted cancer-stem-cell-related characteristics, supporting its use as a tumor model.	Drug-free model; primarily one TNBC cell line and no in vivo validation.
Arumugam et al., 2021 [116]	Cellulose acetate/silk fibroin hybrid nanofibers containing Au–Ag nanoparticles	Au–Ag nanoparticles	NR	MCF-7 and MDA-MB-231	Au–Ag-containing hybrid nanofibers exhibited substantial cytotoxicity against breast cancer cells.	Release behavior of the metallic component was not established; in vitro evaluation only.
Prieto et al., 2021 [117]	Aligned, porous, collagen-coated PCL nanofibers	None	N/A	MCF-7 and MDA-MB-231	Increased breast cancer stem-cell populations, mesenchymal-marker expression, and invasiveness, providing an ECM-mimicking 3D tumor model.	Modeling rather than therapeutic delivery platform; restricted to in vitro studies.
Suárez et al., 2022 [118]	Uniaxial/coaxial PCL/alginate and PCL/gelatin nanofibers	Tamoxifen (TMX) + curcumin (Cur)	TMX showed relatively rapid release, whereas Cur-containing systems provided more prolonged release	MCF-7; PBMCs used for comparison	Nanofibers enabled controlled Cur/TMX delivery and produced marked cytotoxicity against MCF-7 cells.	Some formulations also showed toxicity toward PBMCs; in vitro study only.
Darbasizadeh et al., 2021 [56]	Coaxial PEO/PCL core–sheath nanofibers	Doxorubicin (DOX)	Initial release followed by prolonged, diffusion-controlled release over several weeks	MCF-7	DOX-loaded core–sheath fibers provided prolonged local drug delivery and strong anticancer activity.	In vitro evaluation only; incomplete drug release over the study period.
Rasouli et al., 2020 [119]	PLGA/PEG electrospun nanofibers	Curcumin + chrysin	Sustained/prolonged co-release	T47D	Co-delivery produced synergistic antiproliferative effects and showed potential for preventing local breast cancer recurrence.	In vitro only; recurrence prevention was not demonstrated in an animal model.
Asgari et al., 2021 [120]	Pullulan nanofibers incorporating GO-PCH-g-HPG nanocarriers	Paclitaxel (PTX) + curcumin (Cur)	Burst during the first ~5 h followed by sustained release for ~90 h	MCF-7	Maintained the synergistic anticancer effect of PTX and Cur while enabling localized co-delivery.	In vitro only; the unloaded nanocarrier also exhibited some cellular toxicity.
Ataollahi and Larypoor, 2022 [121]	PVA/lentinan electrospun nanofibers	Docetaxel + lentinan	NR	MCF-7	Combined lentinan/docetaxel treatment produced synergistic anticancer activity and reduced HER3 expression.	Release kinetics were not adequately reported; in vitro model only.
Jouybari et al., 2019 [57]	Triaxial chitosan/PVA–PLA-based multilayer nanofibers containing g-C_3_N_4_	5-Fluorouracil + DOX + PTX	Controlled, sustained multidrug release; 5-FU approached zero-order release behavior	MCF-7	Enabled simultaneous local delivery of three chemotherapeutic agents and produced strong anticancer activity.	In vitro only; complex triaxial fabrication may hinder reproducibility and scale-up.
Mehnath et al., 2020 [122]	Stimuli-responsive core–shell nanofibers containing targeted polymeric micelles	Paclitaxel	pH-responsive, sustained release over several days	MCF-7	Targeted micelle-containing nanofibers enhanced localized PTX delivery and breast-cancer-cell cytotoxicity.	Mainly in vitro evidence; in vivo antitumor efficacy and long-term safety require evaluation.
Akpan et al., 2020 [123]	PLGA/gelatin nanofibers, with/without Pluronic F127	Prodigiosin	Initial burst followed by sustained release over approximately 3 days	MCF-7 and MDA-MB-231	Prodigiosin-loaded fibers produced sustained localized delivery and anticancer activity, including against TNBC cells.	In vitro study; initial burst release remained, and in vivo efficacy was not investigated.
Akpan et al., 2021 [124]	PLGA/gelatin electrospun fibers, with/without Pluronic F127	Annona muricata leaf extract	pH-dependent initial release followed by sustained release	MCF-7 and MDA-MB-231; MCF-10A control	Released extract preferentially inhibited breast cancer cells and promoted apoptotic effects.	In vitro only; botanical extract composition may introduce batch-to-batch variability.
Abasalta et al., 2021 [125]	Coaxial carboxymethyl chitosan/PCL core–shell nanofibers containing nickel ferrite	Doxorubicin	Controlled, pH- and magnetic-field-responsive release	MCF-7	Magnetic core–shell nanofibers enabled controlled DOX delivery and enhanced cytotoxicity under magnetic-field exposure.	Single-cell-line in vitro study; magnetic-field requirement increases treatment complexity.
Hsu et al., 2021 [126]	PLGA electrospun nanofibers	Paclitaxel	Continuous and prolonged PTX release compatible with metronomic chemotherapy	4T1 TNBC cells and orthotopic murine model	Local sustained PTX delivery significantly suppressed TNBC progression and demonstrated the feasibility of implantable metronomic chemotherapy.	Preclinical animal model; clinical dosing, degradation, and long-term implant safety remain to be established.
Babadi et al., 2022 [127]	PCL/collagen electrospun nanofibers	Piperine	Sustained release extending for approximately 16 days	MCF-7 and 4T1; 4T1 tumor-bearing mice	Piperine-loaded fibers suppressed tumor growth and increased apoptosis, supporting postoperative localized therapy.	Preclinical study; human pharmacokinetics and long-term implant safety remain unknown.
Talimi et al., 2023 [128]	Coaxial PCL/chitosan-containing core–shell nanofibers	Sirolimus	Prolonged release from coaxial fibers compared with monoaxial systems	MCF-7 and 4T1; 4T1 tumor-bearing mice	Sirolimus-loaded coaxial fibers inhibited cancer-cell proliferation and reduced tumor growth in vivo.	Preclinical evidence only; long-term degradation and clinical implant performance remain untested.
Seyhan et al., 2023 [129]	PLA/PEG electrospun nanofibers	Amygdalin/bitter almond-derived active material	Controlled release extending to approximately 10 h	MCF-7	Amygdalin-loaded fibers produced dose-dependent cytotoxicity against breast cancer cells and were proposed for prevention of local recurrence.	Short release duration; one cell line and no in vivo efficacy study.
Alabrahim and Azzazy, 2024 [130]	PCL electrospun nanofibers	Pistacia lentiscus essential oil + 5-FU	Sustained release for 168 h, with greater release at acidic pH	MCF-7 and MDA-MB-231; MCF-10A control	Co-loaded fibers showed stronger anticancer activity than either free agent or singly loaded systems, indicating potential synergistic activity.	In vitro study; in vivo therapeutic efficacy and mechanism of synergy remain to be established.
Ding et al., 2016 [131]	PDLLA electrospun nanofibers	Docetaxel (DTX)	Sustained local release	4T1 cells; BALB/c mice after primary breast tumor resection	DTX-loaded nanofibers reduced locoregional recurrence after tumor resection. Recurrence was 16.7% in the nanofiber group compared with 88.9% for blank fibers, 75.0% for systemic DTX, 77.8% for local free DTX, and 100% for the untreated control.	Preclinical mouse study; long-term safety and clinical efficacy remain unknown.
Li et al., 2020 [132]	Hierarchical coaxial PEG/PDLLA electrospun ultrafine fiber device	Doxorubicin hydrochloride + disulfiram	Time-programmed dual release: rapid DOX release and sustained disulfiram release	Breast tumor-bearing mice after surgical tumor reduction	The implanted fiber device reduced postoperative tumor recurrence and metastasis by targeting residual tumor cells and tumor invasion.	Preclinical animal study; complex fiber architecture may make manufacturing and scale-up more difficult.

Abbreviations: N/A, not applicable; NR, not reported; ECM, extracellular matrix.

**Table 4:** Polymer-dependent drug release behavior in electrospun systems.

Polymer Type	Burst Tendency	Sustained-Release Features
Hydrophilic (PVA, HEMA)	High	Requires crosslinking/crystallinity
Hydrophobic (PCL, PLA, PLGA)	Low	Extended diffusion-controlled release
Swelling-controlled gels	Moderate	Gel-layer mediated release
Degrading polyesters (PLGA)	Moderate	Multi-phase erosion-based release
Multilayer coatings	Very low	Predictable long-term release

## Data Availability

No new data were created or analyzed in this study. Data sharing is not applicable to this article.

## Abbreviations

The following abbreviations are used in this manuscript:

Abbreviation	Full Term
3DP	Three-dimensional printing
5-FU	5-Fluorouracil
AD-SCs	Adipose-derived stem cells
AFM	Atomic force microscopy
ALG	Alginate
Alg-DA	Alginate dialdehyde
ATR-FTIR	Attenuated total reflectance Fourier-transform infrared spectroscopy
AuNPs	Gold nanoparticles
BAC	Bacterial cellulose
BCL-2	B-cell lymphoma 2
BMP-2	Bone morphogenetic protein-2
BRCA1	Breast cancer gene 1
BRCA2	Breast cancer gene 2
BMSC	Bone marrow stromal cell
CA	Cellulose acetate
CLSM	Confocal laser scanning microscopy
CHT	Chitosan
CIS	Cisplatin
CPPs	Cell-penetrating peptides
CSC	Cancer stem cell
CT	Computed tomography
CXCL12	C-X-C motif chemokine ligand 12
CXCR4	C-X-C chemokine receptor type 4
DCM	Dichloromethane
DMEM	Dulbecco’s Modified Eagle Medium
DMF	Dimethylformamide
DOX	Doxorubicin
DSC	Differential scanning calorimetry
ECM	Extracellular matrix
EDS	Energy-dispersive X-ray spectroscopy
EDX	Energy-dispersive X-ray analysis
EPR	Enhanced permeability and retention
ER	Estrogen receptor
FDA	Food and Drug Administration
FE-SEM	Field emission scanning electron microscopy
FIB-SEM	Focused ion beam scanning electron microscopy
FITC-BSA	Fluorescein isothiocyanate–bovine serum albumin
FTIR	Fourier-transform infrared spectroscopy
GEL	Gelatin
GPC	Gel permeation chromatography
GMP	Good manufacturing practice
GO	Graphene oxide
GO-PCH-g-HPG	Graphene Oxide–Poly(Citric Acid)-g-Hyperbranched Polyglycerol
gp60	Glycoprotein 60
GSH-NFP	Glutathione-modified nanofiber precursor
HA	Hydroxyapatite
HAuCl4	Gold(III) chloride
HDFs	Human dermal fibroblasts
HEMA	Hydroxyethyl methacrylate
HER2	Human epidermal growth factor receptor 2
HER2/ERBB2	Human Epidermal Growth Factor Receptor 2/Erb-B2 Receptor Tyrosine Kinase 2
HFIP	Hexafluoroisopropanol
HIF-1α	Hypoxia-inducible factor 1 alpha
HPLC	High-performance liquid chromatography
HP-β-CD	Hydroxypropyl beta-cyclodextrin
HR-TEM	High-resolution transmission electron microscopy
ICH	International Council for Harmonisation
ICP-MS	Inductively coupled plasma mass spectrometry
IL	Interleukin
LDH	Lactate dehydrogenase
MBC	Metastatic breast cancer
MC	Indomethacin
MCF-10A	Michigan Cancer Foundation-10A Cell Line
MCF-7	Michigan Cancer Foundation-7 Cell Line
MoS_2_	Molybdenum disulfide
MRI	Magnetic resonance imaging
MTT	3-(4,5-Dimethylthiazol-2-yl)-2,5-Diphenyltetrazolium Bromide
NaGdF4	Sodium gadolinium fluoride
NET	Neutrophil extracellular trap
NETosis	Neutrophil extracellular trap formation
NF	Nanofiber
NFs	Nanofibers
NGF	Nerve growth factor
NHDF	Normal human dermal fibroblasts
NIH	National Institutes of Health
PAD4	Peptidyl arginine deiminase 4
PBS	Phosphate-buffered saline
PCD	Polycyclodextrin
PCL	Polycaprolactone
PCM	Phase-change material
PDO	Polydioxanone
PEG	Polyethylene glycol
PEO	Polyethylene oxide
PET	Positron emission tomography
PHBV	Poly(3-Hydroxybutyrate-co-3-Hydroxyvalerate)
PLA	Poly(lactic acid)
PLGA	Poly(Lactic-co-Glycolic Acid)
PLLA	Poly(L-Lactic Acid)
PR	Progesterone receptor
PTX	Paclitaxel
PU	Polyurethane
PVA	Polyvinyl alcohol
PVOH	Polyvinyl alcohol
PVP	Polyvinylpyrrolidone
qRT-PCR	Quantitative reverse transcription polymerase chain reaction
RES	Reticuloendothelial system
RH	Relative humidity
ROS	Reactive oxygen species
RPMI	Roswell Park Memorial Institute Medium
SEM	Scanning electron microscopy
Smad	Small mothers against decapentaplegic
SPARC	Secreted protein acidic and rich in cysteine
SRB	Sulforhodamine B
STED	Stimulated emission depletion microscopy
TCL	Tetracycline
TEM	Transmission electron microscopy
TFE	Trifluoroethanol
TFV	Tenofovir
TGA	Thermogravimetric analysis
TiO_2_	Titanium dioxide
TNBC	Triple-negative breast cancer
UCNPs	Upconversion nanoparticles
UCL	Upconversion luminescence
UV-Vis	Ultraviolet–visible spectroscopy
VEGF	Vascular endothelial growth factor
WAKO^®^	Wako Pure Chemical Industries assay kit
WST	Water-soluble tetrazolium salt
XPS	X-ray photoelectron spectroscopy
XRD	X-ray diffraction
ZnO	Zinc oxide
